# Birds can transition between stable and unstable states via wing morphing

**DOI:** 10.1038/s41586-022-04477-8

**Published:** 2022-03-09

**Authors:** C. Harvey, V. B. Baliga, J. C. M. Wong, D. L. Altshuler, D. J. Inman

**Affiliations:** 1 Department of Aerospace Engineering, University of Michigan, Ann Arbor, MI USA; 2 Department of Zoology, University of British Columbia, Vancouver, British Columbia Canada

**Keywords:** Biomechanics, Aerospace engineering

## Abstract

Birds morph their wing shape to accomplish extraordinary manoeuvres^1–4^, which are governed by avian-specific equations of motion. Solving these equations requires information about a bird’s aerodynamic and inertial characteristics^5^. Avian flight research to date has focused on resolving aerodynamic features, whereas inertial properties including centre of gravity and moment of inertia are seldom addressed. Here we use an analytical method to determine the inertial characteristics of 22 species across the full range of elbow and wrist flexion and extension. We find that wing morphing allows birds to substantially change their roll and yaw inertia but has a minimal effect on the position of the centre of gravity. With the addition of inertial characteristics, we derived a novel metric of pitch agility and estimated the static pitch stability, revealing that the agility and static margin ranges are reduced as body mass increases. These results provide quantitative evidence that evolution selects for both stable and unstable flight, in contrast to the prevailing narrative that birds are evolving away from stability^6^. This comprehensive analysis of avian inertial characteristics provides the key features required to establish a theoretical model of avian manoeuvrability.

## Abstract (web summary)

Analysis of inertial characteristics across 22 bird species shows that evolution has selected for avian manoeuvrability using both stable and unstable flight dynamics.

## Main

There is currently no theory that provides hypotheses to guide studies of avian manoeuvrability. This is not owing to a lack of physical understanding; manoeuvrability can be broadly defined as a bird’s ability to change the magnitude and direction of its velocity vector^7,8^. Similar to comparable uncrewed aerial vehicles, a bird’s flight dynamics and thus its manoeuvrability are dictated by its governing equations of motion. For example, aircraft dynamics depend on a minimum of six equations, three translational and three rotational, that can be derived from Newton’s second law and its rotational counterpart^5,9^:

1 $${\bf{F}}=\frac{{\rm{d}}(m{\bf{v}})}{{\rm{d}}t}$$

2 $${\bf{M}}=\frac{{\rm{d}}({\bf{I}}{\boldsymbol{\omega }})}{{\rm{d}}t}$$

Where **v** is velocity vector and **ω** is the angular velocity vector. These equations can be combined to solve for a flyer’s acceleration (translationally: $\frac{{\rm{d}}{\bf{v}}}{{\rm{d}}t}$ and rotationally: $\frac{{\rm{d}}{\boldsymbol{\omega }}}{{\rm{d}}t}$), but this requires knowledge of both the aerodynamically informed external forces (**F**) and moments (**M**) as well as the inertial characteristics, including the mass (*m*) and moment of inertia tensor(**I**). However, avian inertial characteristics are not currently available with sufficient breadth or resolution.

**Fig. 1:**
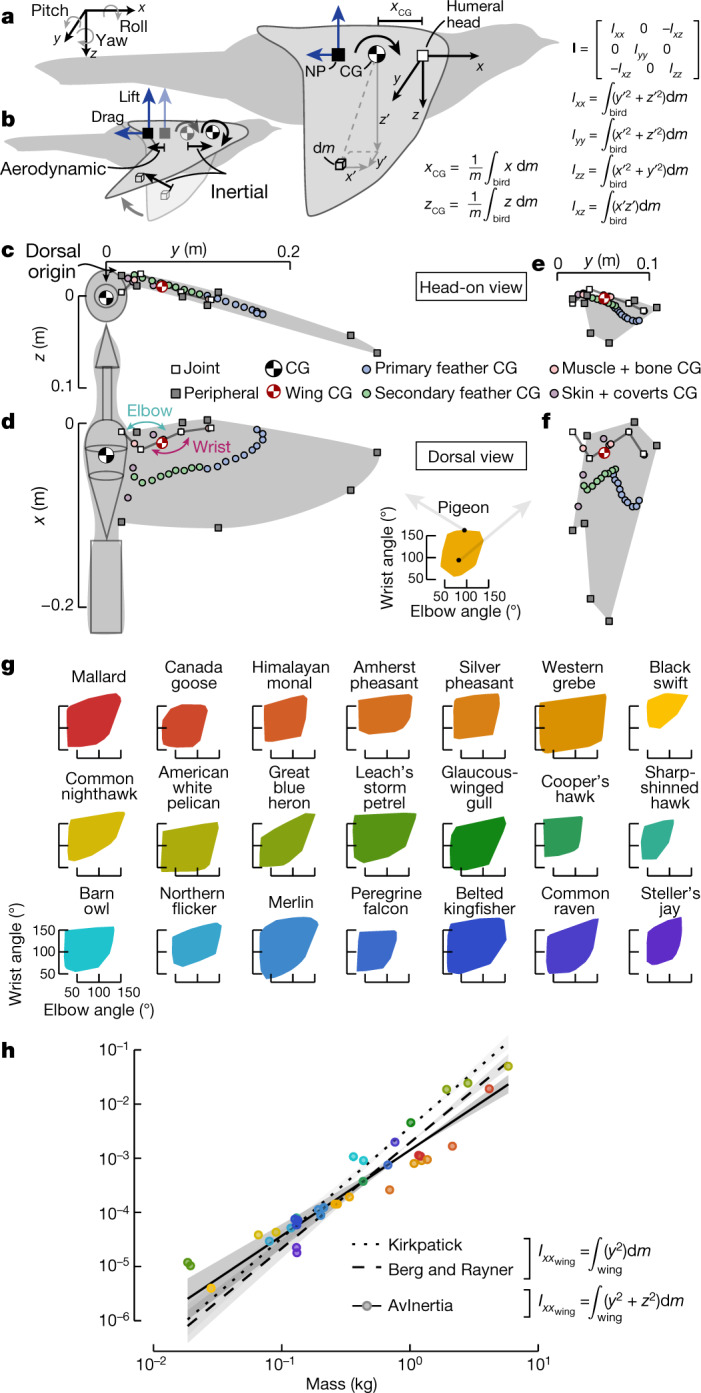
Inertial properties must be determined to quantify avian manoeuvrability. **a**, A bird’s centre of gravity (CG) is the position about which weight is equally distributed, and the neutral point (NP) is where aerodynamic forces can be modelled as point forces and the pitching moment is independent of angle of attack. The moment of inertia (**I**) components are obtained by integrating differential mass elements (d*m*) over the entire bird. **b**, Flight dynamics are affected by adjusting either inertial or aerodynamic characteristics. **c**–**f**, We modelled birds as a composite of simple geometric components. Each component’s centre of gravity varies as a wing morphs from an extended (**c**, **d**) to a folded (**e**, **f**) configuration. **g**, Convex hulls showcase the ROM of the elbow and wrist for 22 species. **h**, The computed maximum ${I}_{{{xx}}_{{\rm{wing}}}}$ was similar to published estimates. *n* = 36 individual specimens; 95% confidence intervals visualized by transparent ribbons.

Therefore, avian flight manoeuvrability is often evaluated experimentally by tracking individuals to measure accelerations during observed manoeuvres^1,3,4^. However, tracking data do not provide a bird’s maximal manoeuvring capabilities or allow extrapolation to unobserved behaviours. Determining these attributes requires a robust and general framework for manoeuvrability, equivalent to the manoeuvrability equations for aircraft^8,10^. Obtaining generalizable data is further complicated because aerodynamic and inertial characteristics vary substantially within and among species, and even dynamically for an individual bird^1,11^. For example, birds can initiate manoeuvres by morphing—that is, changing the orientation and shape of their wings, body and tail^7,12,13^. To progress towards a theoretical formulation of avian manoeuvrability, there has been a marked and justifiable focus on resolving the aerodynamic characteristics of a bird in flight^14–16^. However, studies often overlook the equally essential inertial properties (Fig. 1a) or use static morphology approximations for individual species^13,17–20^. Here we fill this gap by investigating the variable inertial characteristics of flying birds to provide the necessary next step towards establishing a general framework of avian manoeuvrability.

Another challenge to solving a flying bird’s equations of motion is how to properly formulate the equations. For example, the equations can be simplified by defining the origin at the centre of gravity (Fig. 1a), which is equivalent to the centre of mass in a constant gravitational field^9^. If the centre of gravity moves substantially relative to the body, additional terms in the equations are required to properly capture flight dynamics^10^. Physically shifting a bird’s morphology shifts the centre of gravity, but it is not known how much the centre of gravity moves as a bird morphs. In addition, the rotational inertia—quantified by the mass moment of inertia tensor (**I**) about the origin—is also affected by morphing (Fig. 1a, b). This symmetric matrix describes the body mass distribution, where diagonal elements quantify the distribution relative to the major axes (*I*_*xx*_, roll; *I*_*yy*_, pitch; and *I*_*zz*_, yaw) and off-diagonal elements quantify distribution within the three major geometric planes^9^ (only *I*_*xz*_ is non-zero for symmetric configurations; Fig. 1a).

We calculated a bird’s centre of gravity and **I** to evaluate avian manoeuvrability through the lens of agility and static stability. Agility encompasses a bird’s ability to perform linear accelerations (axial agility) and angular accelerations (torsional agility)^7^, and depends on both the centre of gravity^5^ and **I**. In contrast, static stability refers to the initial tendency to return towards an equilibrium after a disturbance^14^. We quantified static pitch stability with the static margin, which is the distance between the centre of gravity and neutral point^5,16^ (Fig. 1a). If the neutral point is behind the centre of gravity, the static margin will be positive and thus stable. Often, stability is inversely related to agility because larger manoeuvring forces and moments are sometimes necessary to overcome stabilizing forces and moments^14^.

To determine how inertial characteristics vary during wing morphing, we developed a general analytical method to quantify any flying bird’s centre of gravity and **I**, and used a comparative analysis to investigate 22 species spanning the phylogeny defined by Prum et al.^21^, except for Palaeognathae as this clade contains largely flightless birds. First, we measured geometric and mass properties of cadavers and used motion tracking on cadaveric wings to extract the range of extension and flexion for the elbow and wrist (Fig. 1g). We limited our study to solely investigate the role of wing morphing due to elbow and wrist flexion and extension because previous studies have shown that this range of motion (ROM) enables a substantial shift in the neutral point^14,16^. The investigated ROM defines a bird’s physical capability to adjust its inertial characteristics and includes wing configurations outside of those probably used in flight. In addition, we assumed that the shoulder was set to allow a comparable wing orientation (see Methods) and that the tail is furled, but these degrees of freedom have an important role in avian flight control^22^ and warrant future morphing studies. Finally, we developed an open-source R package (AvInertia) that models birds as a composite structure of simple geometric objects and uses morphological data to calculate the centre of gravity and **I** for any bird using any wing configuration (Fig. 1c–f, Methods). We validated this methodology with previous static wing measurements (Fig. 1h, Methods).

## Centre of gravity is relatively constant

**Fig. 2:**
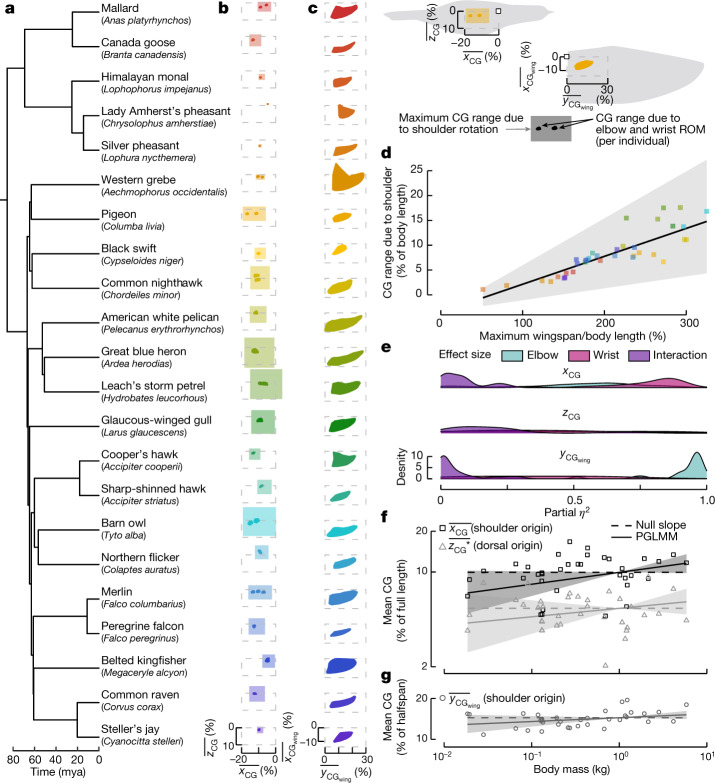
A bird’s centre of gravity is minimally affected by elbow and wrist flexion and extension. **a**, Time-calibrated phylogeny for 22 species (mya, million years ago). **b**, **c**, The elbow and wrist ROM (opaque polygons, convex hulls) affect ${x}_{\bar{{\rm{C}}}{\rm{G}}}$ and ${z}_{\bar{{\rm{C}}}{\rm{G}}}$ (over bar indicates normalization by body length) (**b**) and ${y}_{{\bar{{\rm{C}}}{\rm{G}}}_{{\rm{w}}{\rm{i}}{\rm{n}}{\rm{g}}}}$(over bar indicates normalization by maximum half span) (**c**). **b**, The centre of gravity range is overlaid with the maximum bounds due to 90° shoulder rotation (transparent polygons), (**d**) which increase with increasing ratio of wingspan to body length. **e**, Effect size (partial *η*^2^) of elbow, wrist, and interaction on each centre of gravity component per specimen. **f**, **g**, The log-transformed mean values of ${x}_{\bar{{\rm{C}}}{\rm{G}}}$ and ${z}_{\bar{{\rm{C}}}{\rm{G}}}$* (*denotes the *z* position relative to the dorsal origin defined by Fig. 1c) (**f**) and ${y}_{{\bar{{\rm{C}}}{\rm{G}}}_{{\rm{w}}{\rm{i}}{\rm{n}}{\rm{g}}}}$ did not scale with body mass as the phylogenetic generalized linear mixed model (PGLMM) (solid line) did not differ significantly from the null slope (dashed line). *n* = 36 individual specimens; 95% confidence intervals visualized in **d**, **f**, **g** by transparent ribbons.

With our validated results, we first tested the effect of the elbow and wrist ROM on a bird’s centre of gravity when its wings are held symmetrically. We found that the ROM had a minimal effect on the position of the centre of gravity (Fig. 2b, opaque polygons). The maximum shifts along the *x*-axis and *z*-axis (${x}_{\bar{{\rm{C}}}{\rm{G}}}$ and ${z}_{\bar{{\rm{C}}}{\rm{G}}}$; normalized by the full bird’s length—the subscript CG refers to the centre of gravity) were 3% (great blue heron (*Ardea herodias*), 2.0 cm) and 2% (barn owl (*Tyto alba*), 0.7cm), respectively (Fig. 2b). Despite the small magnitude, wrist extension consistently shifted ${x}_{\bar{{\rm{C}}}{\rm{G}}}$ forwards (*P* < 0.002) and the wrist angle explained a high amount of variance in the data leading to a high effect size, quantified by partial eta-squared (*η*^2^)^23^, ^24^. We found that partial *η*^2^ was greater than 0.34 for all species (Fig. 2e). Similarly, elbow extension tended to shift ${x}_{\bar{{\rm{C}}}{\rm{G}}}$ forwards, but its effect size varied across species. Both elbow and wrist extension predominately shifted ${z}_{\bar{{\rm{C}}}{\rm{G}}}$ dorsally, but the magnitude and effect size varied. We could not differentiate the log-transformed mean ${x}_{\bar{{\rm{C}}}{\rm{G}}}$ or ${z}_{\bar{{\rm{C}}}{\rm{G}}}$ position from those expected if birds were simply scaled by preserving all length scales (that is, isometry) (Fig. 2f, Extended Data Table 1).

The small effect of the elbow and wrist on the location of the centre of gravity led us to question whether this would carry over to shoulder joint motion as well. To obtain a conservative estimate, we assumed that wings could rotate about the humeral head by 90° forwards, backwards, up and down (Fig. 2b, transparent squares). This revealed that the maximum $\triangle {x}_{\bar{{\rm{C}}}{\rm{G}}}$ and $\triangle {z}_{\bar{{\rm{C}}}{\rm{G}}}$ shifts were 18% (10.9 cm) for the great blue heron, approximately sixfold greater than that achieved with elbow and wrist morphing alone. Such a large shift in the centre of gravity probably cannot be neglected when formulating the equations of motion. At the other extreme, the Lady Amherst’s pheasant (*Chrysolophus amherstiae*) had a negligible shift of 1% (1.4 cm) with shoulder joint motion. Across the full range of taxa, we found a significant positive relationship between ${x}_{\bar{{\rm{C}}}{\rm{G}}}$ due to shoulder motion and the ratio of maximum wingspan to body length (Fig. 2d, Extended Data Table 1). This trend suggests that proper modelling of flight dynamics for birds with wings substantially longer than their body length will require an estimation of the expected centre of gravity shift to verify whether a fixed centre of gravity is an appropriate assumption.

Although the full bird’s centre of gravity defines its symmetric flight dynamics, the wing-only parameters can give insight into asymmetric configurations. We found that the elbow and wrist ROM caused the centre of gravity of the wing to shift along the *y* axis ($\triangle {y}_{{\bar{{\rm{C}}}{\rm{G}}}_{{\rm{w}}{\rm{i}}{\rm{n}}{\rm{g}}}}$, normalized by the maximum half span) by between 10% (black swift (*Cypseloides niger*)) and 27% (American white pelican (*Pelecanus erythrorhynchos*)) (Fig. 2c), where the most distal ${y}_{{\bar{{\rm{C}}}{\rm{G}}}_{{\rm{w}}{\rm{i}}{\rm{n}}{\rm{g}}}}$ was 28% (western grebe (*Aechmophorus occidentalis*)). Additionally, $\triangle {y}_{{\bar{{\rm{C}}}{\rm{G}}}_{{\rm{w}}{\rm{i}}{\rm{n}}{\rm{g}}}}$ was positively associated with the arm-to-hand wing ratio (Extended Data Table 1), such that birds with longer hand wings than arm wings (like the swift) would have a reduced capacity to shift the wing’s centre of gravity. The centre of gravity shift was largely driven by elbow extension (*P* < 0.001, partial *η*^2^ > 0.51; Fig. 2e) whereas the effect of the wrist varied across species. These results highlight a well-conserved proximal location of the wing centre of gravity across species. Contrary to a previous study^25^, we did not find that the log-transformed mean ${y}_{{\bar{{\rm{C}}}{\rm{G}}}_{{\rm{w}}{\rm{i}}{\rm{n}}{\rm{g}}}}$ differed from isometric expectations (Fig. 2g, Extended Data Table 1).

## Morphing affects lateral inertia

**Fig. 3:**
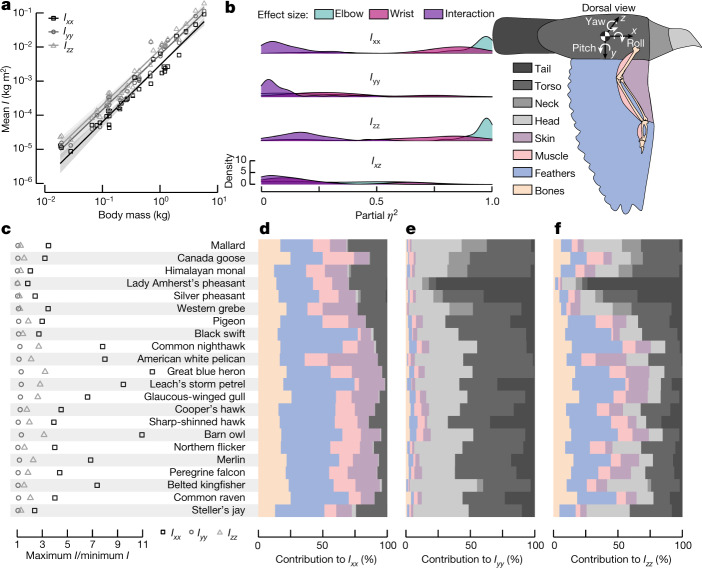
Wing morphing, specifically driven by the elbow, has a strong effect on roll and yaw inertia components. **a**, All log-transformed mean diagonal components scaled isometrically with body mass (PGLMM model for each component; solid line). *n* = 36 individual specimens; 95% confidence intervals visualized by transparent ribbons. **b**, Elbow extension has the largest effect on *I*_*xx*_ and *I*_*zz*_ but joint angles were not strong predictors of *I*_*yy*_ or *I*_*xz*_. **c**, The ability to adjust ***I*** varies substantially across species. **d**–**f**, At the maximum wing extension, the wing components (bones, feathers, muscle and skin) made the largest contribution to *I*_*xx*_ (**d**), whereas body components (head, neck, torso and tail) had a larger role in *I*_*yy*_ (**e**) and *I*_*zz*_ (**f**). Components are coloured following the bird schematic.

The centre of gravity is crucial to formulating the governing equations, but their solution depends on a bird’s rotational inertia. Like the centre of gravity, we found that a bird’s rotational inertia (log-transformed mean diagonal components of **I**) scaled isometrically with body mass (Fig. 3a, Extended Data Table 1). However, we found that elbow and wrist extension provided a more than 11-fold *I*_*xx*_ increase (heron) and a 3-fold *I*_*zz*_ increase (heron and owl) (Fig. 3c). This capability was largely driven by elbow extension (Fig. 3b), which had a significant effect on both *I*_*xx*_ (*P* < 0.001, partial *η*^2^ > 0.23; except for Leach’s storm petrel (*Hydrobates leucorhous*)) and *I*_*zz*_ (*P* < 0.009, partial *η*^2^ > 0.45). The absolute values of *I*_*yy*_ and *I*_*xz*_ were minimally affected by joint extension and the effect size varied substantially across species (Fig. 3b). We next computed the contribution of each major body part to the overall rotational inertia for birds with wings at maximum elbow and wrist extension (Fig. 3d–f). Because the wings were extended along the *y* axis, this captures approximately the lowest wing contribution to *I*_*yy*_ but the highest wing contribution to *I*_*xx*_. The percentage contribution of each body part varied substantially across the species, but as expected the wings were responsible for the majority of *I*_*xx*_. These results indicate that elbow and wrist ROM provides substantial inertial control over the roll and yaw axes (*I*_*xx*,_ *I*_*zz*_), but less so for the pitch axis (*I*_*yy*_), although species-specific differences were also apparent in our results. Incorporating the shoulder joint ROM would increase the wing’s contribution to inertial pitch control.

## Inertia informs the pitch agility metric

**Fig. 4:**
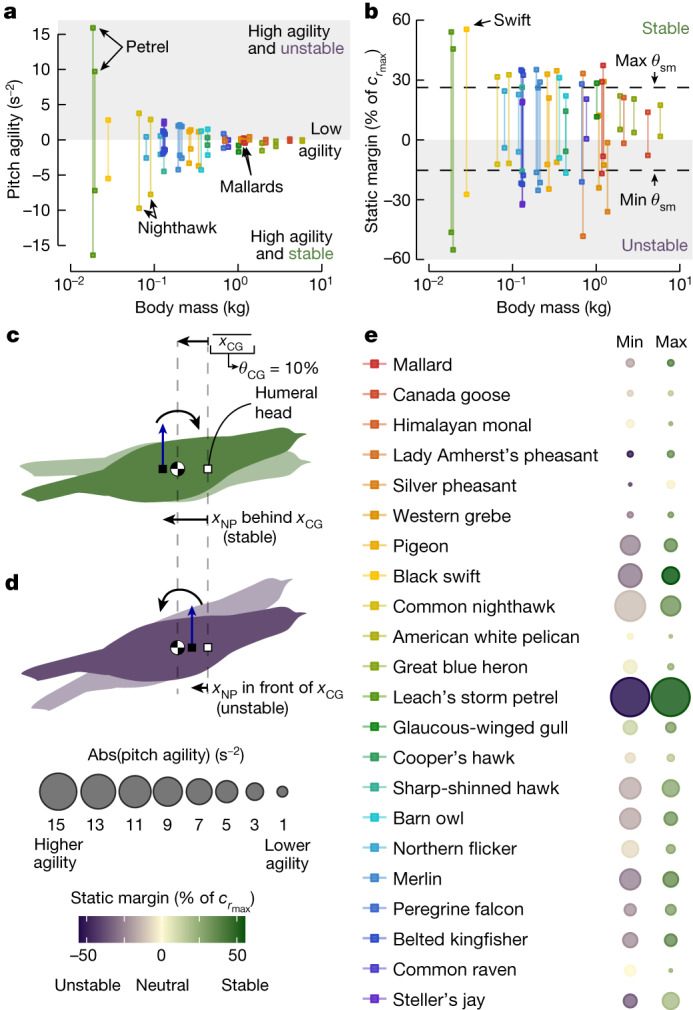
Evolution selects for both pitch stability and instability, but modern birds exhibit highly variable pitch agility and stability characteristics. **a**, **b**, We derived a pitch agility metric, which highlights that heavier birds are less agile (**a**) and have a reduced static margin (sm) range (**b**). Maximum and minimum values for each individual due to elbow and wrist ROM are plotted and the static margin is normalized by each specimen’s maximum root chord (*c*_*r*__max_). An Ornstein Uhlenbeck model provided evidence of selection pressures acting on an unstable minimum (dashed line, Min *θ*_sm_) and a stable maximum (dashed line: Max *θ*_sm_) static margin, and ${x}_{\bar{{\rm{C}}}{\rm{G}}}$ (*θ*_CG_). **c**, **d**, This ${x}_{\bar{{\rm{C}}}{\rm{G}}}$ position is stable if the neutral point is behind this position (**c**) and unstable if the neutral point is in front of this position (**d**). **e**, The investigated species exhibited a wide variety of static margins and absolute pitch agility. Dot colour and size represent the mean maximum and minimum value, respectively, for each species.

We next tested whether inertial characteristics could be used to estimate a bird’s pitch agility. However, because both inertia and aerodynamics are fundamental to flight dynamics, we first used aerodynamic theory and data from a rigid gull wing^16^ to obtain an estimate for the neutral point, and thus the static margin for each configuration (Methods and Supplementary Methods). Using these results, we derived a novel pitch agility metric that is proportional to the angular acceleration about the *y* axis due to a change in the angle of attack (a form of torsional agility; Fig. 1a, Methods). Note that agility in a stable configuration indicates that the developed acceleration would tend to return the bird towards an equilibrium position. We found that the pitch agility range decreases as body mass increases, which was expected because flight speed and body size scale positively with mass^26^ (Fig. 4a, Extended Data Table 1). These results are further driven by the static margin whose range also decreases as mass increases (Fig. 4b, Extended Data Table 1). Incorporating the shoulder joint ROM would broaden the static margin range because the resultant neutral point shift is probably larger than the centre of gravity shift as evidenced by morphing uncrewed aerial vehicles with shoulder-inspired joints^27,28^.

## Evolutionary pressures on stability

**Extended Data Fig. 1:**
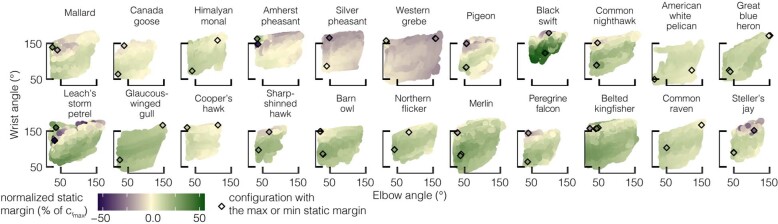
The elbow and wrist angle configurations that yielded the maximum and minimum static margin for each species. The range of motion investigated for each species with the maximum static margin and minimum static margin identified with a black diamond on each species. Note that the diamonds are coloured by the static margin.

**Extended Data Fig. 2:**
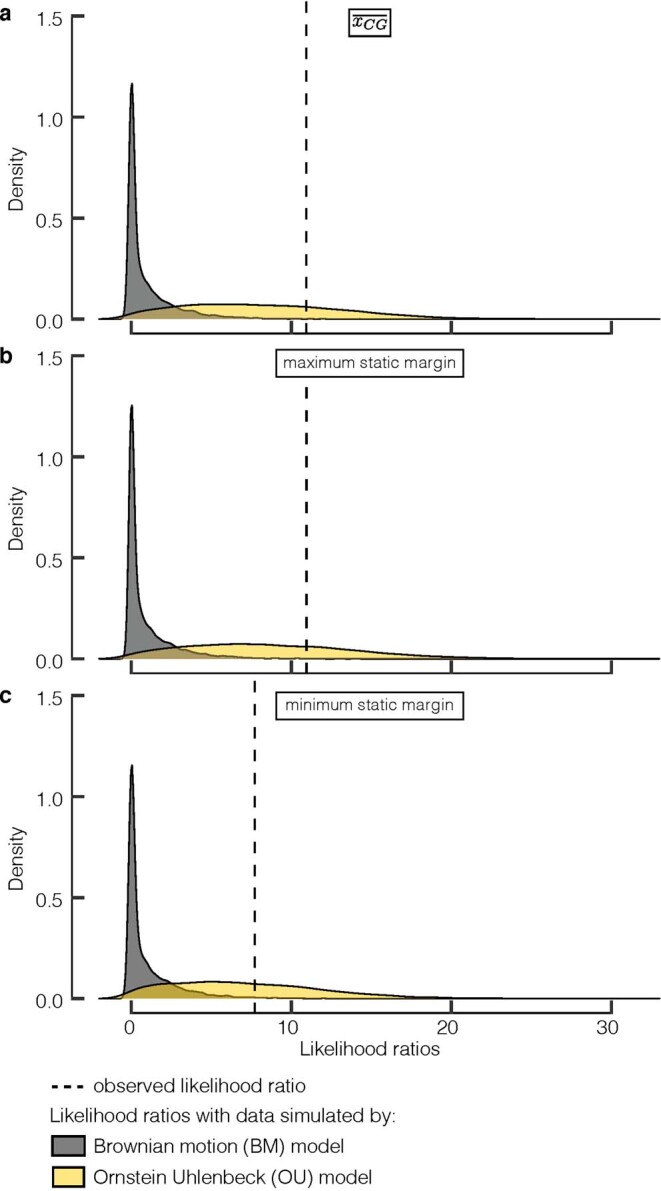
A power analysis confirmed the validity of Ornstein Uhlenbeck models for three key traits. We used a Monte Carlo-based method to investigate if our phylogeny provides support for the use of the Ornstein Uhlenbeck model. This returns the distribution of likelihood ratios under each model for (**a**) ${x}_{\bar{{\rm{C}}}{\rm{G}}}$, (**b**) maximum static margin, and (**c**) minimum static margin. Comparing the likelihood ratio distributions produced under both a Brownian motion (BM) model (grey) and Ornstein Uhlenbeck (OU) model (yellow) to the observed likelihood ratio (dashed black line) revealed that the Ornstein Uhlenbeck model was a better fit for our three key traits (see Methods).

**Extended Data Fig. 3:**
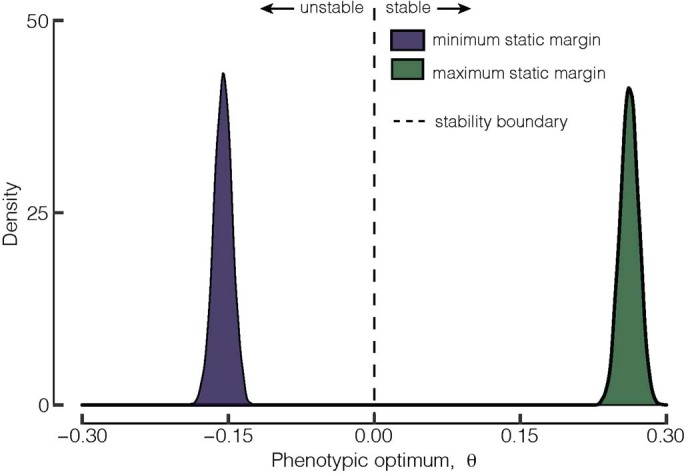
Bootstrapping our results within the conservative centre of gravity  measurement error supported our results. We used a Monte Carlo method to investigate the impact of centre of gravity measurement error on the phenotypic optima for the maximum static margin (green) and minimum static margin (purple). Our results for a stable maximum and an unstable minimum are confirmed with over a 95% confidence interval.

**Extended Data Fig. 4:**
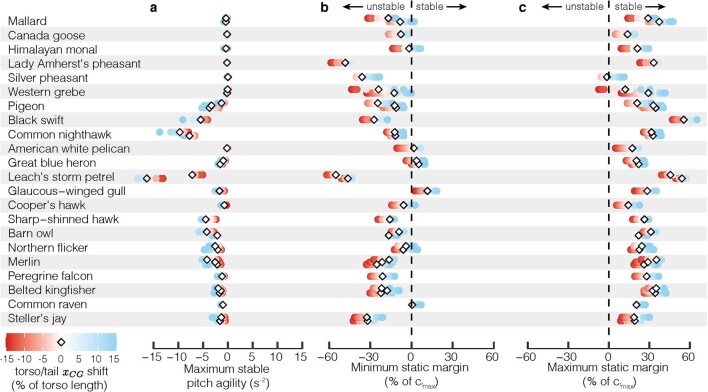
Conservative measurement sensitivity analysis revealed a minimal effect on pitch stability and agility metrics. We assumed that experimental error on the centre of gravity measured for the torso and tail was ±15% of the torso length (up to a maximum of 4 cm) and recalculated the (**a**) maximum pitch agility for stable flight (most negative values in Fig. 4a), (**b**) minimum and (**c**) maximum static margin. The estimated error ranges from panels **b** and **c** informed the bootstrapping analysis in Extended Data Fig. 3.

Next, we looked for evidence of selective evolutionary pressures on avian pitch agility and stability. We investigated the static margin specifically because it is both a component of the pitch agility metric and dictates the static stability of a flying bird. We identified the configurations with the maximum and minimum static margin for each individual (Extended Data Fig. 1) and then calculated the mean of each trait for each species (Fig. 4b). We found that four species were entirely stable, one species was entirely unstable, and 17 species had the capacity to shift between stable and unstable flight (Fig. 4b, e). Using these data, we found that an Ornstein Uhlenbeck model was significantly favoured over a Brownian motion model for both the maximum (ΔAICc = −8.24; Extended Data Fig. 2b) and minimum static margin (ΔAICc = −5.01; Extended Data Fig. 2c), where AICc is the Akaike information criterion with correction for smaller sample sizes. Further, we found that the optimal static margin phenotype (*θ*_sm_) was stable for the maximum static margin (26% of the maximum root chord, strength of selection (*α*_OU_) = 0.53, variance (*σ*^2^) = 14.2 × 10^−3^), whereas the optimal phenotype for the minimum static margin was unstable (−15% of the maximum root chord, *α*_OU_ = 0.06, *σ*^2^ = 2.7 × 10^−3^) (Fig. 4b). This suggests that evolutionary pressures act to maintain birds’ ability to transition between stable and unstable flight. The strength of selection (*α*_OU_) was relatively low, but our results were robust to measurement errors (Extended Data Figs. 3, 4) and to a preliminary estimation of a neutral point shift due to the tail (Supplementary Methods). Further, an Ornstein Uhlenbeck model was a good fit for the mean ${x}_{\bar{{\rm{C}}}{\rm{G}}}$such that the phenotypic optimum (*θ*_CG_) was 10% of the body length behind the humeral head (ΔAICc = −8.23, *α*_OU_ = 0.11, *σ*^2^ = 0.1 × 10^−3^; Extended Data Fig. 2a). The stability of this centre of gravity position depends on the location of the neutral point (Fig. 4c, d).

Although studies have suggested that modern birds may be capable of stabilized flight^13,14,16^, it is widely believed that birds have evolved to be unstable in pitch to enhance manoeuvrability^6^. Our results offer a new perspective on the evolution of avian flight: evolutionary pressures may be maintaining the ability to shift between stable and unstable configurations. Elbow and wrist flexion and extension alone offer birds the capacity to shift between these pitch stability modes but, if and when a flying bird does shift between these modes remains to be seen. As highlighted by Thomas and Taylor^13^, dynamically switching between stable and unstable modes probably requires substantially different control algorithms, and thus switching between these modes would necessitate a complex flight control system. Further, our findings offer insight on how birds perform slow glides with positive tail lift^29^. By maintaining the capacity to relocate the wing–body neutral point in front of the centre of gravity, birds may achieve an equilibrium—albeit unstable—flight condition.

It is important to highlight that further work is required to incorporate the inter- and intra-specific aerodynamic capabilities, shoulder and tail ROM, and in vivo configurations to definitively confirm the optimal phenotype(s) for static pitch stability. We expect that the shoulder joint will enhance the available pitch control and the ability to shift between modes owing to an increased static margin range; the extent of this enhancement will depend on each species’ shoulder ROM^5,27,28^. Future work is also required to extend this analysis to the roll and yaw axes to discuss lateral agility and stability, which will need to account for aerodynamic and inertial coupling^5^. Finally, 23% of the species in our study were unable to shift between stable and unstable modes with the elbow and wrist alone, and thus there are many combinations of stability characteristics in modern birds.

## Conclusions

In summary, our results reveal that elbow and wrist ROM have a small relative effect on the centre of gravity location and pitch inertia, but have a substantial effect on the roll and yaw inertia. Although inter- and intra-specific variation is apparent, we found that the measured range of wrist and elbow motion alone is sufficient to enable switching between stable and unstable flight in 17 out of 22 bird species. Further, an evolutionary analysis shows that the phenotypic optimum maximum and minimum static margin supports the ability to transition between stable and unstable flight, suggesting the need for a complex flight control system. Collectively, investigating the inertial characteristics of flying birds throughout elbow and wrist ROM brings us a step closer to establishing a fundamental theory to quantify and evaluate avian manoeuvrability.

## Methods

### Collection of morphological data

We obtained morphological data for 36 adult specimens representing 22 species (Fig. 2a) from frozen cadavers acquired from the Cowan Tetrapod Collection at the Beaty Biodiversity Museum (University of British Columbia, Vancouver, Canada). Sample size was a function of the availability and quality of specimens from the museum as we could only rely on fully-intact, well-preserved specimens. The cadavers were inspected to ensure adequate condition and completeness, after which we measured the full body mass, wingspan, and body length. Next, we disarticulated the wing at the shoulder joint, taking care to ensure that each wing’s skin, propatagial elements, and feathers remained intact. One wing from each cadaver was used to determine wing ROM and corresponding wing shape change (see ‘Determination of the elbow and wrist ROM’). The cadaver was further dissected to obtain length and mass measurements for the head, neck, torso, wing components, legs, and tail (refer to Supplementary Methods for details on each measurement). We obtained the centre of gravity coordinates for the torso (body without head, neck, tail, wings) by manually balancing the torso and measuring the distance from the clavicle reference point to the balanced position. Note that because of the preservation of the storm petrel specimens, we estimated the mass on the basis of humerus bone length and the torso centre of gravity as being proportional to that of the gull. Finally, we individually weighed and photographed each flight feather, enabling geometric parameters to be extracted using ImageJ software^30^. Refer to the publicly available data for details on all assumptions used for extracting the morphological measurements. Note that this study consisted of a single experimental group and thus randomization and blinding was not necessary.

### Determination of the elbow and wrist ROM

To determine the wing ROM and corresponding shape change, we actuated the cadaver wings throughout the full range of extension and flexion of the elbow and wrist joints by hand (following methods established by Baliga et al.^11^, Fig. 1g). We tracked the location of 10 reflective markers each 4 mm in diameter (grey and white points in Fig. 1c–f, refer to Supplementary Methods for details) with automated 3D data capture at 30 frames per second using a 4- or 5-camera tracking system (OptiTrack, NaturalPoint). Using tools from NaturalPoint, each recording was calibrated to have less than 0.5 mm overall mean reprojection error. Joint angles were calculated as the interior angle defined by three key points: points 1, 2 (vertex) and 3 for the elbow, and points 2, 3 (vertex) and 4 for the wrist (Supplementary Methods).

### Developing AvInertia

We developed an open source R package (AvInertia) to calculate the centre of gravity and moment of inertia tensor (**I**) for any flying bird (Fig. 1a) in RStudio^31^ (version 1.3.1093) running R^32^ (version 4.0.3). A high-level overview of the code methodology follows in this section. Further details are provided in the Supplementary Methods, as each individual component of the avian models required specific procedures and approximations.

To allow a generalized approach, we used a common methodology from mechanics to estimate the centre of gravity and inertia components using simple geometric shapes^9^. We elected to use as many elements as possible to allow the best resolution. For each species, we first modelled the bird’s body without the wings as a composite of five components: head, neck, torso, legs and tail. To determine the inertial properties of the wings, we aligned each wing configuration extracted from the ROM measurements so that the wrist joint was in line with the shoulder joint along the *y* and *z* axes and so that the wrist joint was aligned with the first secondary feather (S1) along the *x* axis (extended wing: Fig. 1c, d; folded wing: Fig. 1e, f). Note that this positioning results in a different shoulder angle between each wing configuration and wings with extremely low elbow angles and high wrist angles being positioned at substantially different incidence angles than the body. Each wing was then modelled as a composite of twelve components: bones (humerus, radius, ulna, carpometacarpus/digit, radiale and ulnare), muscles (brachial, antebrachial and manus groups), skin, coverts, and tertiary feathers. In addition, each primary and secondary feather was modelled and positioned individually as a composite structure of five components: calamus, rachis (cortex exterior and medullar interior), and distal and proximal vanes. AvInertia permits a variable number of flight feathers. With our methodology, a bird with 10 primaries and 10 secondaries that flies with an extended neck will be represented by a composite model with 232 individual simple geometric shapes. In our study, we investigated only symmetric wing configurations for a full bird and considered the effects of a single wing independently. We assumed that anisotropic effects such as the air space within the body would have a minimal impact on the overall centre of gravity^33^.

To calculate the final inertial characteristics of this composite bird, each component’s shape, mass, and positioning was informed by its corresponding morphological measurements. We began by determining the centre of gravity and **I** for one of the basic geometric shapes with respect to an origin and frame of reference that simplified the formulation of the centre of gravity and **I** for that shape. Next, AvInertia computed the mass-weighted summation of the centre of gravity of each object and shifted the origin to the bird reference point, located at the centre of the spinal cord when cut at the clavicle. The centre of gravity was then transformed into the full bird frame of reference, which is defined by Fig. 1c–f. We used the parallel axis theorem and the appropriate transformation matrices to transform **I** to be defined about the final centre of gravity within the full bird frame of reference.

### Validating AvInertia

We validated our methodology by comparing the maximum rotational inertia about the roll axis for a single wing (${{I}_{{xx}}}_{{\rm{wing}}}$, origin at the humeral head) to data from previous experimental studies that measured ${{I}_{{xx}}}_{{\rm{wing}}}$ by cutting an extended wing into strips^25,34^ (Fig. 1h). Our 95% confidence intervals on the exponent of body mass marginally overlapped with Berg and Rayner’s predictions^25^ but were significantly lower than Kirkpatrick’s predictions^34^. However, Kirkpatrick used 10 wing strips while Berg and Rayner later found that at least 15 strips were necessary to minimize systematic error^25,34^. Next, we directly compared results for the pigeon (*Columba livia*), the only species in common between the studies, and found ${{I}_{{xx}}}_{{\rm{wing}}}$(×10^4^) was between 1.42 and 1.92 kg m^2^, which encompasses values from previous studies^25,34,35^ (1.72 and 1.83 kg m^2^). The pigeon wing’s maximum centre of gravity position along the *y*-axis (${y}_{{\bar{{\rm{C}}}{\rm{G}}}_{{\rm{w}}{\rm{i}}{\rm{n}}{\rm{g}}}}$) was only 3% of the half span more proximal than Berg and Rayner’s measurement^25^. We expect minor differences because strip methods enforce that all wing mass is contained within the *x–y* plane while AvInertia accounts for out-of-plane morphology (Fig. 1h).

### Agility and stability metrics

We developed a pitch agility metric that estimates the change of the angular acceleration about the *y* axis ($\triangle \dot{q}$, known as the time rate of change of the pitch rate) due to a degree change in the angle of attack (Δ*α*) as:

3 $$\frac{{\rm{\bigtriangleup }}\dot{q}}{{\rm{\bigtriangleup }}\alpha }\propto \frac{\left[\left({\left(\frac{{\mathop{x}\limits^{ \sim }}_{c/4}}{{c}_{{r}_{max}}}\right)}^{0.8}{c}_{{r}_{max}}\right)-{x}_{{\rm{C}}{\rm{G}}}\right]{({m}^{0.12})}^{2}{S}_{max}}{{I}_{yy}}$$

Where *m* is the body mass, *c*_*r*__max_ is the maximum root chord for the specimen, *S*_max_ is the maximum single wing area for the specimen, *x*_CG_ is the centre of gravity position on the *x*-axis measured from the humeral head, and ${\widetilde{x}}_{c/4}$ is the quarter chord of the standard mean chord^37^ (defined in equation (7)). This equation was derived beginning from the rigid aircraft *y* axis rotational equation of motion assuming a symmetric configuration undergoing small disturbances^5^:

4 $$\triangle M={I}_{{yy}}\triangle \dot{q}$$

From this equation, we estimated the change in pitching moment (Δ*Μ*) with a Taylor series expansion method assuming that the largest effect is due to angle of attack and then non-dimensionalized as follows^5^:

5 $$\Delta M=\frac{{\rm{\partial }}M}{{\rm{\partial }}\alpha }\Delta \alpha =\frac{1}{2}\rho {V}^{2}(2{S}_{max}){c}_{{r}_{max}}\frac{{\rm{\partial }}{C}_{M}}{{\rm{\partial }}\alpha }\Delta \alpha =\frac{1}{2}\rho {V}^{2}(2{S}_{max}){c}_{{r}_{max}}\frac{{\rm{\partial }}{C}_{M}}{{\rm{\partial }}{C}_{L}}\frac{{\rm{\partial }}{C}_{L}}{{\rm{\partial }}\alpha }\Delta \alpha $$

Where *ρ* is air density, *V* is the freestream scalar velocity, and *C*_*M*_ and *C*_*L*_ are the coefficients of pitching moment and lift, respectively. Because the pitching moment slope $(\frac{{\rm{\partial }}{C}_{M}}{{\rm{\partial }}{C}_{L}})$ is proportional to static margin^16,36^, we estimated each configuration’s neutral point (indicated by the subscript NP) using our previous morphing gull wing-body aerodynamic results (see Supplementary Methods). This analysis revealed that the neutral point for a wing-body configuration scaled with:

6 $$\,\frac{{x}_{{\rm{NP}}}}{{c}_{{r}_{{\rm{\max }}}}}\approx {\left(\frac{{\widetilde{x}}_{c/4}}{{c}_{{r}_{{\rm{\max }}}}}\right)}^{0.8}$$

${\widetilde{x}}_{c/4}$ is the quarter chord of the standard mean chord defined as^37^:

7 $${\widetilde{x}}_{c/4}=\frac{{\int }_{0}^{b/2}c(\,y\,){x}_{c/4}(\,y\,){\rm{d}}y}{{\int }_{0}^{b/2}c(\,y\,){\rm{d}}y}$$

Where *b* is the wingspan and, *c* and *x*_*c/4*_ are the chord and quarter chord location as a function of the span position (*y*), respectively. This equation was evaluated numerically for each of the bird wings modelled with 1,000 segments. In addition, we performed a sensitivity analysis on the exponent (see Supplementary Methods). With the estimated neutral point, we calculated the static margin as:

8 $${\rm{S}}{\rm{t}}{\rm{a}}{\rm{t}}{\rm{i}}{\rm{c}}\,{\rm{m}}{\rm{a}}{\rm{r}}{\rm{g}}{\rm{i}}{\rm{n}}=-\frac{{\rm{\partial }}{C}_{M}}{{\rm{\partial }}{C}_{L}}=\frac{{x}_{CG}-\left({\left(\frac{{\mathop{x}\limits^{ \sim }}_{c/4}}{{c}_{{r}_{max}}}\right)}^{0.8}{c}_{{r}_{max}}\right)}{{c}_{{r}_{max}}}$$

Refer to Supplementary Methods for further details pertaining to the aerodynamic assumptions.

For the pitch agility metric, we incorporated a previously established allometric scaling^26^ of cruise velocity ($V\propto$ *m*^0.12^). We assumed a constant air density (*ρ*) and constant lift slope $(\frac{{\rm{\partial }}{C}_{L}}{{\rm{\partial }}\alpha })$ across species to obtain the final proportional relationship as:

9 $${\rm{\bigtriangleup }}M\propto \left[\left({\left(\frac{{\mathop{x}\limits^{ \sim }}_{c/4}}{{c}_{{r}_{max}}}\right)}^{0.8}{c}_{{r}_{max}}\right)-{x}_{{\rm{C}}{\rm{G}}}\right]{({m}^{0.12})}^{2}{S}_{max}{\rm{\bigtriangleup }}\alpha $$

This result was then returned to equation (4) and rearranged to obtain the pitch agility metric as seen in equation (3).

### Phylogenetic and statistical analyses

All phylogenetically informed analyses were carried out using the time-calibrated maximum clade credibility tree from Baliga et al.^11^, which was pruned to the 22 focal taxa in this study. To determine the linear trends with body mass, we fit first-order phylogenetic generalized linear mixed models (PGLMM) to the data using the R package MCMCglmm^38^ where the random effects are informed by the phylogeny (Extended Data Table 1). Note that the linear trend of the pitch agility range with body mass remains significant even if the storm petrels are removed from the data. All PGLMM models had priors specified with the inverse Wishart scaling parameters **V** = 1 and *ν* = 0.02 and used 1.3 × 10^7^ Markov chain Monte Carlo iterations. As visualized by Fig. 2f, we cannot definitively exclude the possibility that the lower 95% confidence interval on ${x}_{\bar{{\rm{C}}}{\rm{G}}}$ may be positive which would indicate that ${x}_{\bar{{\rm{C}}}{\rm{G}}}$ scales greater than isometric predictions. However, multiple MCMCglmm runs returned an insignificant result. To determine the significance and effect of the elbow and wrist on the centre of gravity and **I** components, we independently fit first order interactive models to each specimens’ data with a constant scaling on the elbow and wrist angle. We calculated the effect size of the elbow and wrist using the R package effectsize^23^ and independently fit first order interactive models to each specimens’ data with scaled and mean centred elbow and wrist angles.

Next, to investigate the phenotypic optimum of the pitch agility and stability traits$,$ we independently fit both Brownian motion and Ornstein Uhlenbeck models to the absolute data using the R package geiger^39^. We assumed that all species belong to the same regime and thus, fit single-peak evolutionary models. This analysis revealed that there was evidence that the Ornstein Uhlenbeck model was a better model fit for all three of our selected traits (${x}_{\bar{{\rm{C}}}{\rm{G}}}$ maximum and minimum static margin) due to a lower Akaike information criterion with correction for smaller sample sizes (AICc). Because of the smaller sample size of our study^40^, we ran a Monte Carlo simulation (*n* = 5,000) with the R package pmc^41^ to validate that selecting the Ornstein Uhlenbeck model over the Brownian motion model was appropriate (Extended Data Fig. 2). This method returns a distribution of likelihood ratios (twice the difference of the maximum log likelihood for each model) when the traits have been simulated *n* times under each model. These distributions are then compared to the observed likelihood ratio (black dashed vertical lines in Extended Data Fig. 2). For details, refer to Boettiger et al.^41^. We found that the likelihood ratio predicted by a Brownian motion model was more extreme than the observed ratio for the minority of simulations (${x}_{\bar{{\rm{C}}}{\rm{G}}}$:0.2%, maximum static margin: 0.1%, minimum static margin: 1%). Further we had sufficient power to differentiate the two models as the majority of the simulations under the Ornstein Uhlenbeck model fell outside of 95th percentile of the Brownian motion distribution (${x}_{\bar{{\rm{C}}}{\rm{G}}}$ :73.8%, maximum static margin: 77.2%, minimum static margin: 67.2%). 95% confidence intervals were constructed for each reported metric of each trait (Extended Data Table 2). Together these results provide confidence that the observed likelihood ratio of each trait is more likely to occur under an Ornstein Uhlenbeck model than a Brownian motion model.

### Sensitivity analysis

Because both the pitch agility and stability metrics directly depend on ${x}_{{\rm{C}}{\rm{G}}}$, we investigated the sensitivity caused by shifting the combined torso and tail centre of gravity forwards and backwards by up to 15% of the torso. Note that for some species there was a physical limit to the ability to relocate the centre of gravity while maintaining the known morphological properties and if the shifted distance was larger than 4 cm we removed it from the analysis as that was assumed to be an overestimate. The final estimated shift of the relative maximum and minimum static margin is shown in Extended Data Fig. 4. This sensitivity analysis revealed a minor effect on the parameters.

Finally, we wanted to investigate the potential effect of error in our measured centre of gravity metric on our key evolutionary results. To this end, we used a custom bootstrapping code (*n* = 5,000) and randomly sampled (with replacements) from each specimen’s centre of gravity error range used for the sensitivity analysis to recalculate the mean value of the minimum and maximum static margin for each species. With each of these new trait distributions, we re-fit an Ornstein Uhlenbeck model and extracted the optimal phenotype (Extended Data Fig. 3). We found that even allowing for this substantial centre of gravity error, all minimum static margin cases had an unstable optimum and all maximum static margin cases had a stable optimum (Extended Data Fig. 3). Note that this analysis is equivalent to both accounting for the same magnitude shift in the neutral point with a fixed centre of gravity as well as accounting for possible inter-specific variation within the error bounds shown in Extended Data Fig. 4.

### Reporting summary

Further information on research design is available in the Nature Research Reporting Summary linked to this paper.

## Online content

Any methods, additional references, Nature Research reporting summaries, source data, extended data, supplementary information, acknowledgements, peer review information; details of author contributions and competing interests; and statements of data and code availability are available at 10.1038/s41586-022-04477-8.

### Supplementary information

Supplementary Methods This file contains all Supplementary Methods pertaining to the aerodynamic analysis and implementation of AvInertia and associated Supplementary Figs. 1–7 and Supplementary Table 1.

Reporting Summary

Peer Review File

## Figures and Tables

**Extended Data Table 1:**
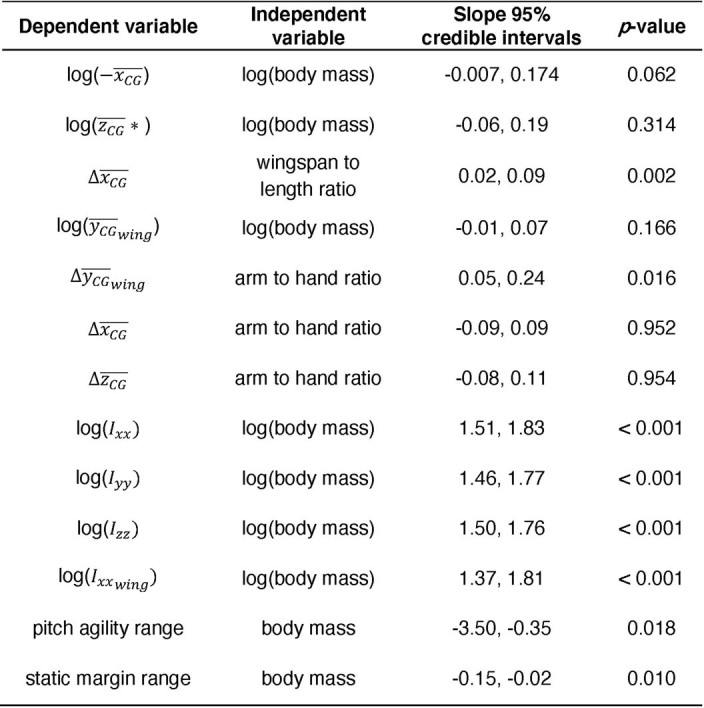
MCMCglmm outputs for all phylogenetic generalized linear mixed models (PGLMM)

Note that all log transforms use the natural base to compare to isometric predictions. Further, the first two models required the inputs to be positive and thus we used the negative of the ${x}_{\bar{{\rm{C}}}{\rm{G}}}$ and defined ${z}_{\bar{{\rm{C}}}{\rm{G}}}*$ to be the *z* position relative to the dorsal origin defined by Fig. 1c. All models involved two-sided tests of their respective hypotheses and *p*-values are presented without controlling for multiple comparisons.

**Extended Data Table 2:**
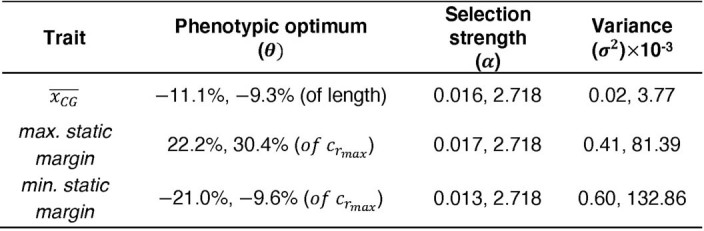
95% confidence intervals on the Ornstein Uhlenbeck metrics reported for each investigated trait

## Data Availability

All data used in this study have been deposited in public repositories identified at 10.6084/m9.figshare.c.5503989.

## Code availability

AvInertia has been publicly released on GitHub (https://github.com/charvey23/AvInertia) and as a CRAN package (https://cran.r-project.org/package=AvInertia). All analysis codes used in this study can be accessed from the public repositories identified at 10.6084/m9.figshare.c.5503989.
