# Supplementary material for: Birds can transition between stable and unstable states via wing morphing
Source: Nature. 2022 Mar 9;603(7902):648–53. doi: 10.1038/s41586-022-04477-8 (PMC8942853; doi:10.1038/s41586-022-04477-8)
Supplement: Supplementary file 1 — This file contains all Supplementary Methods pertaining to the aerodynamic analysis and implementation of AvInertia and associated Supplementary Figs. 1–7 and Supplementary Table 1. [file 41586_2022_4477_MOESM1_ESM.pdf]

nature portfolio

https://doi.org/10.1038/s41586-022-04477-8

Supplementary information

Birds can transition between stable and unstable states via wing morphing

In the format provided by the authors and unedited

Supplemental methods to *Birds can transition between stable and unstable states via wing morphing*

C. Harvey[1,*], V.B. Baliga[2], J.C.M. Wong[2], D.L. Altshuler[2], and D.J. Inman[1]

[1]Department of Aerospace Engineering, University of Michigan, Ann Arbor, MI, USA 48109
[2]Department of Zoology, University of British Columbia, Vancouver, BC, Canada V6T 1Z4
[*]harveyca@umich.edu

ABSTRACT

This document contains the supplemental methods relating to the aerodynamic approximations and the implementation of *AvInertia* that we used for the paper entitled "Birds can transition between stable and unstable states via wing morphing".

Contents

1 Aerodynamics - Estimation of the wing-body neutral point 2

2 Aerodynamics - Estimation of the neutral point shift due to a furled tail 4

3 *AvInertia* - General Procedure 6

High level methodology • Required measurements • Assumptions

4 *AvInertia* - Wing Approximations 7

4.1 Bones . . . 7

Methodology • Required measurements • Assumptions

4.2 Muscles . . . 8

Methodology • Required measurements • Assumptions

4.3 Flight feathers . . . 8

Methodology • Required measurements • Assumptions

4.4 Tertiaries and skin/coverts . . . 11

Methodology • Required measurements • Assumptions

5 *AvInertia* - Rest of the bird approximations 11

5.1 Head, neck, legs and tail . . . 11

Methodology • Required measurements • Assumptions

5.2 Torso . . . 12

Required measurements • Assumptions

References 17

List of Figures

1 **Tail volume coefficients as a function of the body mass for the investigated specimens.** . . . 5

2 **Dorsal view of entire bird modeled as a composite of simplified geometric shapes.** . . . 6

3 **Simplified bone diagram including the referenced frames of reference.** . . . 7

4 **Simplified feather diagram including the referenced frames of reference.** . . . 9

5 **Entire bird measurements.** . . . 15

6 **Wing specific measurements.** . . . 15

7 **Body specific measurements.** . . . 16

List of Tables

1 **Linear fit results for each chord metric.** 4

Glossary

b	wingspan
c_m	mean chord
c_r	wing root chord, i.e. $c(0)$
$c_{r_{max}}$	maximum wing root chord for one specimen
$c(y)$	wing chord as a function of the span position
$C_{L\alpha_t}$	lift-slope of the tail (accounting for interaction effects)
$C_{L\alpha}$	lift-slope of the wing-body configuration
l_t	absolute distance between the flyer's CG and the tail's aerodynamic centre
MAC	mean aerodynamic centre
NP	neutral point of the full flyer
NP_{wb}	neutral point of the wing-body configuration
S_t	total tail area
S_w	total wing area
V_H	tail volume coefficient
x	position along the x axis, chordwise
$x_{c/4}$	position of the quarter chord along the x axis
$\tilde{x}_{c/4}$	position of the quarter chord of the standard mean chord along the x axis
y	position along the y axis, spanwise
y_{cen}	y position of the wing's centroid
α	angle of attack of the full flyer
ε	downwash angle due to the wing
η	dynamic pressure ratio at the horizontal tail

1 Aerodynamics - Estimation of the wing-body neutral point

To estimate the neutral point of a bird's wing-body configuration, we leveraged our previous study on rigid gull wing-body configurations across the *in vivo* range of motion of elbow and wrist flexion and extension[1]. In this previous study, we extracted the neutral point of the wing-body configurations by fitting a linear model to the change of the pitching moment with the lift force[2,3] and provided the morphological information about the associated wing shapes. We used the same wing orientation as the current work. The aerodynamic results were calculated using a numerical lifting line model which was validated with wind tunnel tests on 3D printed wings. As it was not feasible to replicate this analysis for each species in this study, we investigated if there was a metric that could be used to appropriately estimate the neutral point using morphology alone. We assumed that the bird's neutral point could be approximated by the wing-body configuration neutral point. This approximation is appropriate if the wing produces the majority of the lift as is expected with a furled tail[4], but Section 2 discusses the implications of incorporating the tail.

Our approach was informed by traditional aerodynamic theory which predicts that the aerodynamic centre of a 2D thin airfoil will be at the quarter-chord location[5], this work can be extended to lifting line theory for steady flight conditions (commonly used in gliding flight) or blade element theory for revolving airfoils (commonly used in flapping flight). However, 2D thin airfoil approximations do not hold for the thick airfoils known to be used on the proximal wing sections of bird wings[6] nor for 3D wing shapes[5]. Although advances in analytical methods have resulted in mathematical relationships that account for constant taper ratios or sweep[7,8], little information exists for wing shapes as complex as bird wings who have substantial and often nonlinear distributions of geometric twist, taper, sweep, and dihedral. Therefore, we investigated six different chord-based metrics[2,8,9] to establish which would best approximate the neutral point. The origin for all metrics within this study was set at the shoulder joint. This investigation included:

1. **Root chord.** A simplistic approach that estimates the neutral point to be at the quarter chord of the root chord. This approach cannot account for a neutral point shifting forwards of the root leading edge.

2. **Mean chord.** The mean chord is first found as[8]:

$$c_m = \frac{S_w}{b}, \tag{1}$$

Next, we located the most interior span section that had a chord equal to c_m. At that span section we extracted the leading edge position along the x axis and added it to the quarter chord of c_m while accounting for any wing twist at this span section. This final value was taken as the quarter chord position of the mean chord.

3. **Mean projected chord.** Similar approach as the mean chord but the wing area used is that calculated by projecting the wing periphery onto the x-y plane.

4. **Mean aerodynamic chord.** Similar approach as the mean chord but the mean aerodynamic chord is calculated as[8]:

$$MAC = \frac{\int_0^{b/2} c(y)^2 dy}{\int_0^{b/2} c(y) dy}, \tag{2}$$

This value is calculated numerically for the wings by disctretizing the wing along the y axis into 1000 segments. All integral equations that follow used the same discretization.

5. **Centroid area chord.** In this approach we first numerically calculated the position of the wing's centroid along the y axis as[8]:

$$y_{cen} = \frac{\int_0^{b/2} c(y) y dy}{\int_0^{b/2} c(y) dy} \tag{3}$$

Then, at this span location we calculated the chord of the wing. Similar to the approach in the mean chord we determined the quarter chord and leading edge position.

6. **Standard mean chord.** This method allows use to numerically calculate the quarter chord position directly as[8]:

$$\tilde{x}_{c/4} = \frac{\int_0^{b/2} c(y) x_{c/4}(y) dy}{\int_0^{b/2} c(y) dy}, \tag{4}$$

Finally, we normalized each of the output quarter chord positions from the six different metrics by the maximum root chord ($c_{r_{max}}$) of the specimen. This normalization ensures that the result could be scaled for different sized individuals. To assess the fit of each normalized metric to the gull's measured neutral point we fit log-transformed linear models in R:

$$\ln\left(\frac{|x_{NP_{wb}}|}{c_{r_{max}}}\right) \sim \mathbf{A} \ln\left(\frac{|x_?|}{c_{r_{max}}}\right) + \mathbf{B}. \tag{5}$$

Note that for all configurations investigated in our previous study, the neutral point had a negative position on the x axis (aft of the shoulder joint), which allowed us to take the absolute value of the data. Surprisingly, the best fit to our data was the standard mean chord (metric 6) as shown by a low model offset (B) and higher adjusted R^2. The other mean chord parameters (metrics 2 and 3) were a particularly bad fit to our data because often the mean chord was located distally so folding the wrist caused a substantial aftward shift of the estimated quarter-chord location. All model fit parameters are listed in Table 1. With this information, we calculated the relationship for each other species in our study with the exponent of 0.8 and assuming that $e^{-0.052} = 0.949 \approx 1$ as:

$$\frac{|x_{NP_{wb}}|}{c_{r_{max}}} = \left(\frac{|\tilde{x}_{c/4}|}{c_{r_{max}}}\right)^{0.8}. \tag{6}$$

We imported this relationship into our comparative analysis and computed the quarter chord position of the standard mean chord for each specimen and configuration. Next, we checked if the quarter-chord position was in front of the shoulder and if so we switched the signs of the output. This essentially reflects the exponential trend that was established for the gull wings into the positive neutral point region.

We verified that our key static margin findings were not substantially affected by the selected exponent by performing a sensitivity analysis. We found that despite varying the exponent from 0.7 to 1.1, all the optimal phenotypes for maximum static stability were stable whereas the optimal phenotypes for minimum static stability were unstable. Specifically, as the scaling parameter increased, we found that the optimal phenotype for the maximum static margin shifted from 31% to 15% of the maximum root chord and the minimum static margin shifted from -13% to -19%. Thus, increasing the exponent effectively serves to shift the results towards instability but our evolutionary findings remain supported. As the gull represents the only

Supplemental Table 1 | Linear fit results for each chord metric.

Chord metric name	Metric	A (95% CI)	B (95% CI)	Adjusted R^2
root	1	0.833 (0.764, 0.902)	-0.997 (-1.015, -0.980)	0.358
mean projected	2	0.083 (0.062, 0.105)	-1.102 (-1.127, -1.077)	0.053
mean	3	0.054 (0.033, 0.074)	-1.115 (-1.145, -1.084)	0.025
mean aerodynamic	4	0.152 (0.135, 0.169)	-0.930 (-0.960, -0.900)	0.235
centroid area	5	1.349 (1.285, 1.414)	1.436 (1.310, 1.562)	0.624
standard mean	6	0.812 (0.789, 0.835)	-0.052 (-0.084, -0.021)	0.832

species with which we are able to estimate the relationship between geometric parameters and the neutral point across the full range of flexion and extension, we have selected to proceed with the gull-informed exponent of 0.8.

In all, this result provides insight into the aerodynamic implications of morphing however this was informed by an aerodynamic analysis of only one species for rigid wing-body configurations. It will be important for future studies to account for inter-specific differences as well as a neutral point shift due to flexibility and porosity. In addition, we assumed that pitching moment caused by the lift force dominates the resultant moment similar in a manner similar to traditional aerodynamic analyses, however this work should be extended in the future to account for the effect of drag as well.

2 Aerodynamics - Estimation of the neutral point shift due to a furled tail

In the previous section, we established a morphological-based metric to estimate the neutral point of the wing-body configuration. The neutral point of the wing-body configuration is assumed to be a close approximation to the total flyer's neutral point because in our study we assumed that the tail is furled and thus produces a minimal lift. In practice, any additional surface area on a flyer will affect its aerodynamic properties. Further, on traditional aircraft the tail is a major contributor to the overall stability due to the tail aerodynamic centre always remaining aft of the centre of gravity. Proper estimation of the tail contribution requires a detailed analysis of the aerodynamics of the tail and body-wing-tail interactions, but it is possible to make a preliminary estimation the tail contribution with standard aerodynamic theory. We used a standard equation[2,3,10] to estimate the tail's effect on the neutral point to the wing-body configurations (NP_{wb}) as:

$$NP = NP_{wb} - \frac{C_{L\alpha_t}}{C_{L\alpha}}\left(1 - \frac{d\varepsilon}{d\alpha}\right)\eta V_H c_m \tag{7}$$

Where the tail volume coefficient is defined as:

$$V_H = \frac{l_t S_t}{c_m S_w} \tag{8}$$

Note that chord (c_m) introduced in equation 8 is canceled out in equation 7. For plotting purposes we selected to use the mean value of all the mean chords calculated at each morphed configuration for each individual specimen. This allows a comparable metric to the traditional aircraft metrics published by Raymer[10]. We estimated that the tail aerodynamic centre was at 25% of the tail length, although future work will need to account for the expected shift due to low tail aspect ratio. Figure 1 shows the estimated tail volume per individual as a function of the overall body mass. The range for each specimen is largely caused by the changes in the total wing area (S_w). Of note, traditional aircraft tail volume coefficients usually vary between 0.4 to 1 and many of these bird tail volumes are substantially lower[10,11].

Next, we must estimate the factors that multiply the tail volume coefficient in equation 7. First, it is known that the dynamic pressure at the tail will be lower than that at the wing due to the resultant wing wake, which indicates that $\eta < 1$[3]. Next, because of the tail will have a substantially lower aspect ratio than the wing while it is furled, we estimated that the lift-slope of the tail will be lower than that of the wing (i.e. $\frac{C_{L\alpha_t}}{C_{L\alpha}} < 1$). Finally, $\frac{d\varepsilon}{d\alpha}$ is also always below one due to the downwash from the main wing[3,10]. For traditional aircraft in subsonic flight, this value decreases as the wing aspect ratio and taper ratio increase and as the distance between the tail aerodynamic centre and the centre of gravity increases[10]. Each of these characteristics varies substantially across bird species. Because $\frac{d\varepsilon}{d\alpha}$ is subtracted from one in equation 7, decreasing $\frac{d\varepsilon}{d\alpha}$ will effectively increase the tail contribution to the neutral point shift. Collectively, because all three discussed multiplying factors are expected to be less than one, we selected each value to be 0.9 for birds: $\frac{C_{L\alpha_t}}{C_{L\alpha}}(1 - \frac{d\varepsilon}{d\alpha})\eta \approx 0.73$. We selected 0.9 as it is expected to overestimate

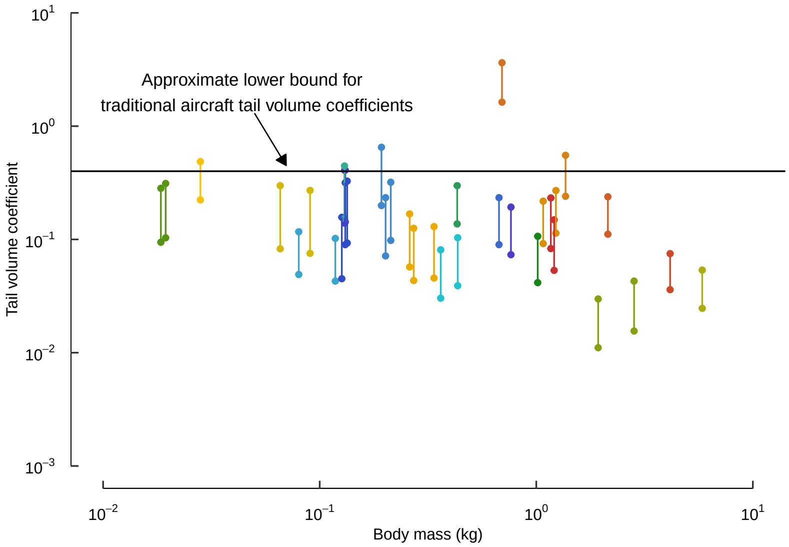

Supplemental Fig. 1 | Tail volume coefficients as a function of the body mass for the investigated specimens.

this multiplying factor. We selected to overestimate this factor because this will return the most aft NP shift and provides a highly conservative view on the validity of our evolutionary results.

With these inputs we solved equation 7 and equation (8) in the main manuscript to obtain the maximum and minimum static margin for each specimen and then calculated the mean value for each species. Next, we fit a Ornstein Uhlenbeck (OU) model to the data following the similar procedure detailed in the methods. This analysis revealed that even when accounting for the tail's effect on the neutral point there is evidence that evolution selects for a stable maximum static margin (max $\theta_{sm} = 38\%$ of $c_{r_{max}}$, $\alpha = 2.718, \sigma^2 = 0.255$) and an unstable minimum static margin (min $\theta_{sm} = -6\%$ of $c_{r_{max}}$, $\alpha = 0.395, \sigma^2 = 0.022$). As expected, the phenotypic optimum values of both the maximum and minimum static margin models shifted towards increased stability, but there was evidence of stronger selection pressure (α) than in the wing-body configurations alone. As with our key results, this could additionally suggest that birds have the ability to shift their neutral point in front of their centre of gravity to balance the positive tail lift that is required for weight support in slow gliding flight[12]. It is important to highlight that we expect inter-specific variation within the multiplying parameters and that the selected value substantially overestimates the tail's contribution and likely results in a more stable output. In all, these results are expected to provide solely a preliminary estimation of the tail's contribution.

3 *AvInertia* - General Procedure

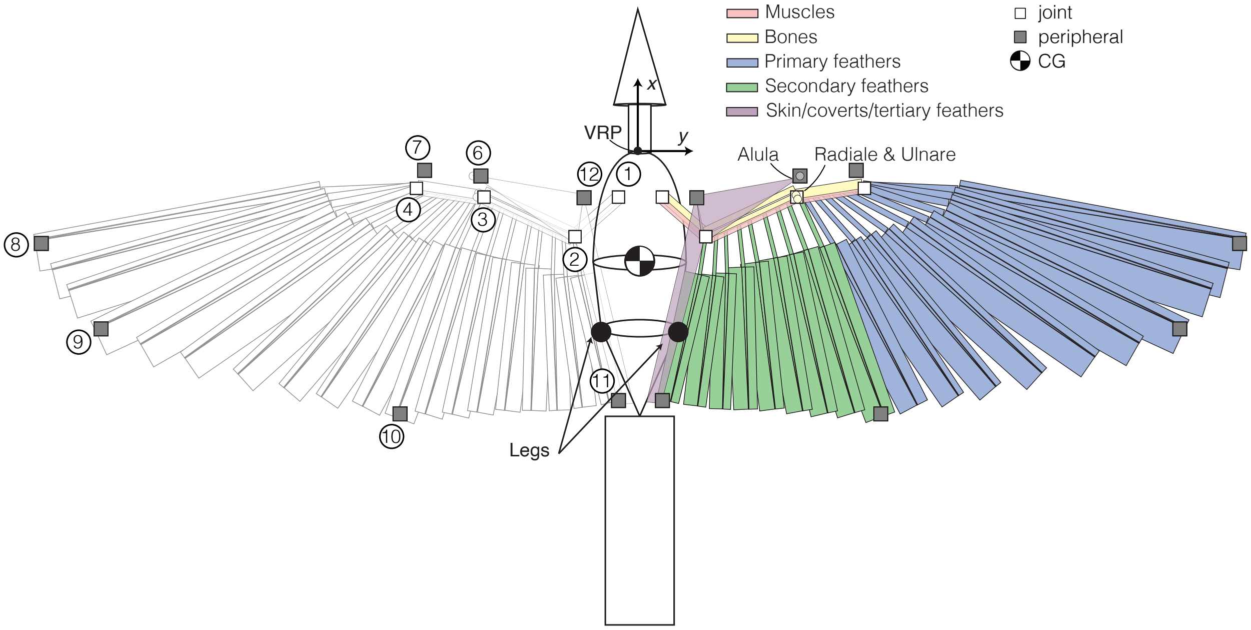

Supplemental Fig. 2 | Dorsal view of entire bird modeled as a composite of simplified geometric shapes.

The final outputs from *AvInertia* are returned in the pre-selected origin and axis system: the bird (vehicle) reference point (VRP) and "full bird" frame of reference (always right-handed axes). In our work, we selected the VRP to be the location where the neck attaches to the torso (Fig. 2) . This is approximately the center of the spinal cord if cut at the clavicle. The x-axis points forwards along the center of the bird, z-axis points ventrally and y-axis points along the right wing. The selection of the origin and axis system is user-specific but must be consistently followed for all inputs. All measurements input into the program should be defined relative to this same origin unless otherwise noted in this document.

Multiple frames of reference are utilized throughout this program. Each individual section will detail the appropriate frame of reference utilized.

3.0.1 High level methodology

1. Model all bird components as a simplified geometric shape.
2. Determine moment of inertia tensor ($\boldsymbol{I}$) and center of gravity (CG) of each component within a frame of reference and about an origin that simplifies their formulation.
3. Transform $\boldsymbol{I}$ and CG to be within the full bird frame frame of reference with the VRP as the origin. This procedure is highly variable and the following sub-sections detail how each component is transformed to be in this final frame of reference.
4. Combine $\boldsymbol{I}$ and CG of each component appropriately.
5. Shift the origin of $\boldsymbol{I}$ to be about the final full CG location.

   Note Parallel axis theorem is only valid between an arbitrary point and the center of gravity, not between two arbitrary points[13]. This was accounted for within the code.

3.0.2 Required measurements

1. Full bird mass, m_{bird}
2. Single wing mass, m_{wing}
3. Position of the wing defined by ten key landmarks (Fig. 2). Note that the identity of Pt11 varies among species; see *"birdmeasurements_readytorun.csv"* in the publicly available data repository for total feather counts. In addition, we do

not include Pt5 and Pt7 as these positions were not needed within the analysis. We did not renumber to avoid confusion.

- Pt1: Humeral head
- Pt2: Center of motion of the elbow joint
- Pt3: Center of motion of the wrist joint
- Pt4: Distal tip of the carpometacarpus
- Pt6: Wing leading edge ahead of the wrist joint
- Pt8: The distal tip of the final primary feather (usually P10)
- Pt9: Distal tip of the fourth-to-last primary feather (usually P7)
- Pt10: Distal tip of the first secondary feather (S1)
- Pt11: Distal tip of the final secondary feather
- Pt12: The most proximal location along the leading edge of the wing

3.0.3 Assumptions

1. **Base geometric shapes** are the greatest assumption within this code although it is commonly used for estimating the inertial characteristics of complex objects[13]. Biological specimens are variable and the accuracy of this assumption will vary between different species.

2. **Wings were aligned** so that the point on the wrist joint is in line with the shoulder along the y axis and along the x axis. The wing was then rotated so that the point on the feather tip at the wing root was at the same height (on the z axis) as the shoulder. However, this is not inherent to *AvInertia* and any wing alignment can be input.

4 *AvInertia* - Wing Approximations

The wing is modeled as a composite structure of bones, muscles, feathers and skin. When computing the final bird inertial properties it is possible to model both symmetric and asymmetric wing shapes. In this work, we focused solely on symmetric configurations.

4.1 Bones

The major wing bones (humerus, radius, ulna and carpometacarpus) are modeled as hollow cylinders with solid end caps[14, 15]. The radiale and ulnare are modeled as point masses at the wrist joint (Pt3). In addition to all physical measurements, we must know the location of the beginning of the bone and the end of the bone to extract the appropriate orientation.

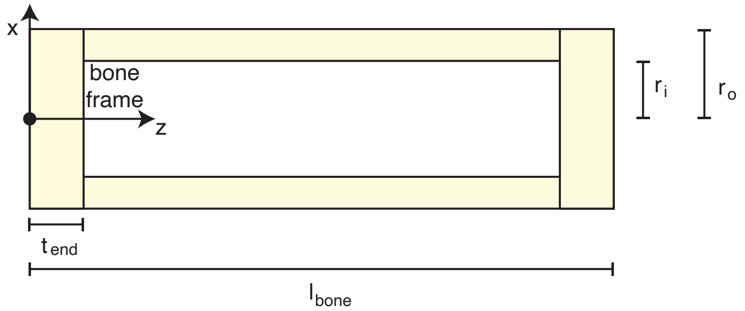

Supplemental Fig. 3 | Simplified bone diagram including the referenced frames of reference.

4.1.1 Methodology

1. Calculate the end cap thickness from:

$$\frac{m_{bone}}{\rho_{bone}} = 2(\pi r_o^2 t_{end}) + \pi({r_o}^2 - {r_i}^2)(l_{bone} - 2t_{end}) \tag{9}$$

2. Calculate the mass of each end cap and the hollow cylinder.

3. Determine $\boldsymbol{I}$ and CG of the hollow cylinder (eqn. 13 and 21) and two end caps (eqn. 14 and 21) with respect to the bone specific frame of reference and origin (Fig. 3).

4. Transform each $\boldsymbol{I}$ and CG to be measured with the VRP as the origin.

5. Sum $\boldsymbol{I}$ of the hollow cylinder and two end caps.

6. Transform $\boldsymbol{I}$ and CG to be expressed within the full bird frame.

7. Radiale and ulnare estimated as point masses on Pt3 within the full bird frame.

4.1.2 Required measurements

1. Mass of each bone, m_{bone}

2. Length of each bone, l_{bone}

3. Average radius of each bone, r_o

4.1.3 Assumptions

1. **Density** (ρ_{bone}) treated as constant 2060 kg/m^3 for all major bones[16].

2. **Inner radius** (r_i) assumed to be 78% of the outer radius[17].

3. **Neglect all other wing bones.**

4. **Carpometacarpus length and mass** include the first digit as well.

5. **CG is at the center of the measured bone length.** This may differ slightly from that extracted using the Optitrack markers. The start of the bone is assumed to be at the most proximal Optitrack marker.

4.2 Muscles

The muscles in the wing are grouped into the brachial, antebrachial and manus regions and assuming that the muscle mass is stretched the length of the major wing bones. In addition to all physical measurements, we must know the location of the beginning of the bone and the end of the bone associated with each muscle group to extract the appropriate orientation.

4.2.1 Methodology

1. Calculate the cylinder radius based on the mass and muscle density for the current group.

2. Determine $\boldsymbol{I}$ and CG of the cylinder (eqn. 14 and 21) within the bone frame of reference and origin (Fig. 3).

3. Transform each $\boldsymbol{I}$ and CG to be measured with the VRP as the origin.

4.2.2 Required measurements

1. Mass of each muscle group, m_{muscle}

2. Length of each bone, l_{bone}

4.2.3 Assumptions

1. **Density** ($\rho_{muscles}$) was assumed to be 1100 kg/m^{-3} for all groups[18]. This is slightly higher than 1060 kg/m^{-3} that was calculated for muscles alone[19] because we did want to include the tendons and connective tissues in the overall calculation.

2. **Muscles are stretched** along the length. In reality, muscles will be more heavily grouped to the start and end section but for simplicity we assumed a constant muscle width along the bone length.

3. **Radius** of the muscles is determined based on the muscle group mass, estimated muscle density and the length of the bones.

4.3 Flight feathers

The flight feathers including all primaries and secondaries are modeled as a composite object (Fig. 4). The calamus is modelled as a hollow cortex cylinder[20], the rachis as a hollow cortex exterior square pyramid and a solid medullary interior square pyramid[20,21], and the vanes as flat rectangular plates. In addition to all physical measurements, we must provide the approximate location of the feather tip and root to extract the appropriate orientation.

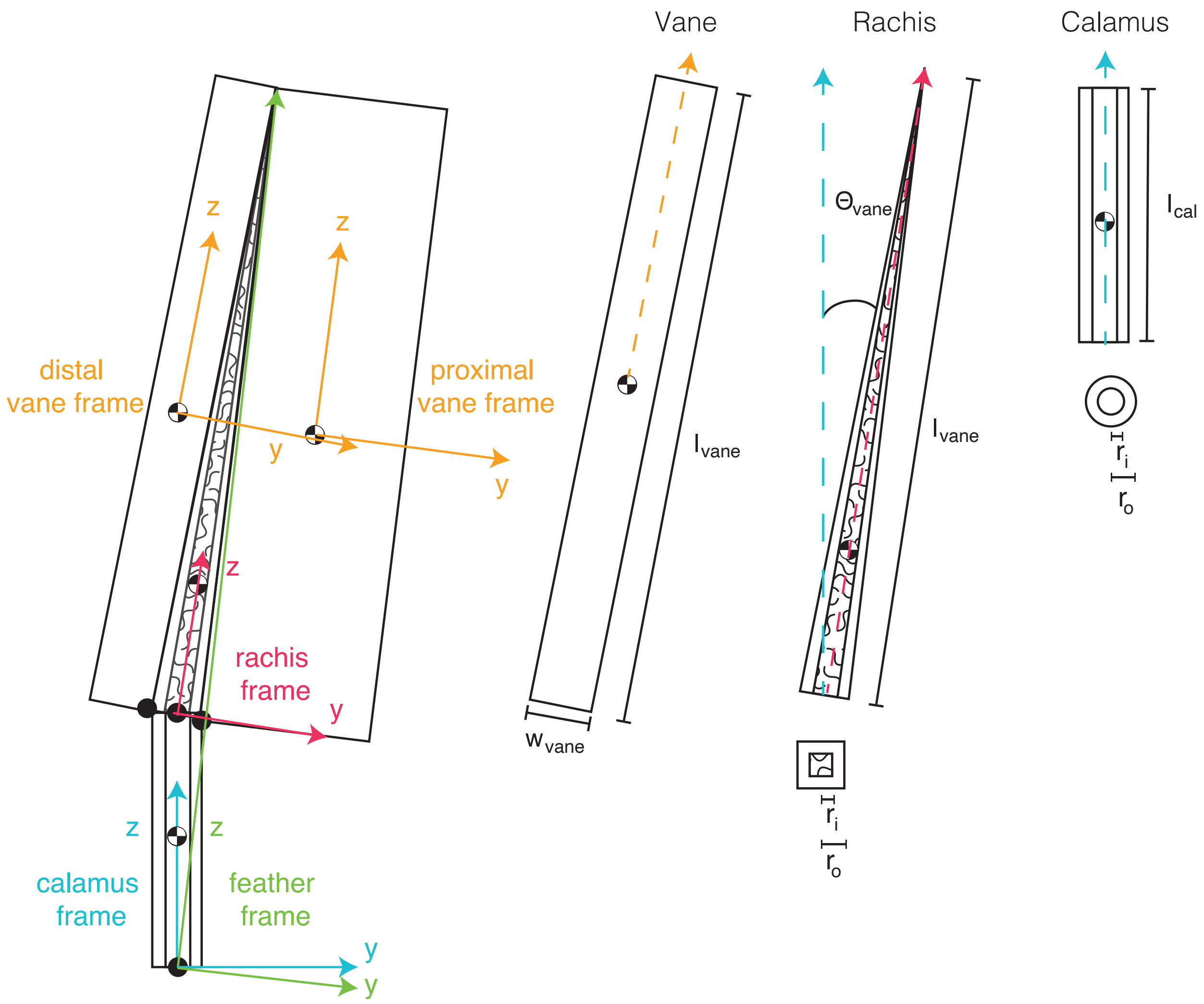

Supplemental Fig. 4 | Simplified feather diagram including the referenced frames of reference.

4.3.1 Methodology

1. Calculate the approximate vane mass assuming vanes are composed of solid cortex cylindrical barbs with a previously determined[22,23] barb radius (r_{barb}) and spacing (d_{barb}) per:

$$n_{barbs} = l_{vane}/(d_{barb} + 2r_{barb}), \tag{10}$$

$$m_{vane} = \rho_{cortex} n_{barbs} w_{vane} \pi r_{barb}^2. \tag{11}$$

2. Substract the proximal and distal vane masses from the total feather mass m_f to determine the total mass of the rachis and calamus (m_{rc}).

3. Calculate the inner radius ($r_{i_{cal}}$) of the calamus and width of the interior rachis pyramid by ensuring that m_{rc} is equal to the mass predicted by the volume and density of the calamus and rachis components:

$$m_{rc} = \rho_{cor}(\pi(r_{o_{cal}}^2 - r_{i_{cal}}^2)l_{cal} + \frac{4}{3}(r_{o_{cal}}^2 - r_{i_{cal}}^2)l_{vane}) + \rho_{med}(\frac{4}{3}r_{i_{cal}}^2 l_{vane}). \tag{12}$$

4. Determine $\boldsymbol{I}$ and CG of the calamus (eqn. 14 and 21) within the calamus frame assuming a hollow cylinder.

5. Determine $\boldsymbol{I}$ and CG of the rachis (eqn. 18 and 22) within the rachis frame assuming exterior cortex and interior medullary retangular pyramids.
6. Determine $\boldsymbol{I}$ and CG of each vane (eqn. 19) within their respective vane frames assuming flat rectangular plates with a mass calculated in Step 1.
7. Transform each vane property into the rachis frame.
8. Combine the rachis and vane $\boldsymbol{I}$ and CG properties.
9. Transform rachis and vane $\boldsymbol{I}$ and CG to be expressed in the calamus frame, (rotate by θ_{vane}) with the origin as the start of the feather.
10. Combine $\boldsymbol{I}$ and CG for the rachis and vane with the calamus components.
11. Transform $\boldsymbol{I}$ and CG to be expressed in the feather frame. Up to this point all of this inertial data can be computed with no knowledge of the current wing positioning. For this reason, the code has two seperate functions relating to the feather inertial calculations.
12. Transform $\boldsymbol{I}$ and CG to be expressed in the full bird frame using information about each individual feather positioning and orientation.
13. Alula feathers estimated as a point mass on Pt6 within the full bird frame.

4.3.2 Required measurements

1. Mass of each feather, m_f
2. Length of the calamus, l_{cal}
3. Length of the vane, l_{vane}
4. Outer radius of the calamus, $r_{o_{cal}}$
5. Distance between barbs, d_{barb}
6. Radius of feather barbs, r_{barb}
7. Average width of proximal and distal vanes, w_{vane}
8. Interior angle between calamus and rachis, θ_{vane}

4.3.3 Assumptions

1. **Density** of cortex (ρ_{cor})[21,24–27] and of medullary (ρ_{med})[25,28] material treated as constant 1150 kg/m^3 and 80 kg/m^3, respectively.
2. **Shape of feathers** was assumed to be constant within a species. We measured the shape properties for only one specimen but, individually measured the mass of each specimen's feathers. Then we assumed isometric scaling to adjust the length and area measurements for each feather as necessary.
3. **Length of medullary** part of the rachis extends all the way to the feather tip[21].
4. **Mass of vanes** is based on previously measured barb radii and distance between barbs[22,23].
5. **Proximal and distal vane barb** properties are treated as constant. Note that previous work did find slight but measurable differences between the vanes that will be neglected in this work[29].
6. **Alula feathers** are treated as a point mass on Pt6 although their structure differs between species.
7. **Feather positioning:**
    - The base of the secondaries are equally spaced along the ulna and their tips are equally spaced along the line between Pt10 and Pt 11 (last secondary).
    - The base of P1 through P6 are equally spaced along the carpometacarpus and their tips are equally spaced along the line between Pt10 and Pt9.
    - The base P7 and up are located at the end of the carpometacarpus (Pt4) and their tips are equally spaced along the line between Pt9 and Pt8.
8. **Feather orientation:**
    - Primaries lay flat on the plane defined by Pt3, Pt4 and their tip position as defined above.
    - Secondaries lay flat on the plane defined by Pt2, Pt3 and their tip position as defined above.

4.4 Tertiaries and skin/coverts

The tertiary feathers and skin/coverts are modeled as flat triangular plates. The tertiary feather sections are defined as two sections, with vertices as follows: 1) Pt12, Pt2 and the trailing edge of the wing at the body and 2) Pt11, Pt2 and the trailing edge of the wing at the body. The skin/coverts section vertices are defined by Pt12, Pt2 and Pt6.

4.4.1 Methodology

1. Given the input positions calculate $\boldsymbol{I}$ and CG based on the general polygon formulations[30] within a frame of reference and about an origin that simplifies their formulation.
2. Transform $\boldsymbol{I}$ and CG to be within the full bird frame and shift so that the VRP is the origin.

4.4.2 Required measurements

1. Mass of the skin and coverts, m_{skin}
2. Mass of the tertiaries, $m_{tertiaries}$

4.4.3 Assumptions

1. **Skin density** (ρ_{skin}) treated as constant (1060 kg/m^3) based on a previously measured muscle-only measurement[19,31]. This was used to calculate the final skin thickness based on ensuring that the volume would return the known mass of the section.
2. **Tertiary density** is treated as constant and equal to the cortex density (ρ_{cor})[21,24–27] of 1150 kg/m^3. As with the skin this was used to calculate the final thickness of the tertiary sections based on ensuring that the volume would return the known mass of the section.
3. **Tertiary mass** is divided equally between the two tertiary sections.

5 *AvInertia* - Rest of the bird approximations

5.1 Head, neck, legs and tail

The head (including the beak) was modeled as a solid cone, neck was modeled as a solid cylinder, legs were point masses and the tail was modeled as a flat rectangular plate.

5.1.1 Methodology

1. Calculate $\boldsymbol{I}$ and CG of the head (eqn. 16 and eqn. 22), neck (if used, eqn. 14 and eqn. 21) and tail (eqn. 19).
2. Calculate $\boldsymbol{I}$ and CG of the legs as point masses placed on the ventral sides of the bird.
3. Transform $\boldsymbol{I}$ and CG to be within the full bird frame and shift so that the VRP is the origin.

5.1.2 Required measurements

1. Mass of the head, m_{head}
2. Length of the head (tip of beak to neck), l_{head}
3. Radius of the head (maximum), r_{head}
4. Mass of the neck, m_{neck}
5. Length of the neck outstretched, l_{neck}
6. Radius of the neck, r_{neck}
7. Mass of the tail, m_{tail}
8. Length of the furled tail, l_{tail}
9. Width of the furled tail, w_{tail}
10. Length of the torso + tail, l_{tot}
11. Mass of both legs, m_{leg}
12. x-location of leg insertion, l_{leg}

5.1.3 Assumptions

1. **Head/beak CG** was measured on the specimens and we found that for all of the measured species the head CG was within 15% of the quarter of the head length (See *"VerificationData.xlsx"* tab *"HeadCGVerification"* in the publicly available data repository). Thus, we assumed that a solid cone would be a fair approximation of the shape.
2. **Neck was only included for some species** that are known to stretch out their neck while in a cruise flight configuration. If not outstretched, the neck mass is added to the head mass. This can be adjusted for general use.
3. **Legs modeled as point masses** however some birds do stick their legs behind their body while in flight. Because grebes have very minimal tails and fly with their legs directly behind their bodies the grebes' legs were treated as a tail. However, for other species this effect was neglected in our work and should be investigated in future studies.

5.2 Torso

The torso is modeled as a structure composed of a hemiellipsoid, partial elliptical cone and either a full elliptical cone (3 individuals) or elliptical cylinder (33 individuals) (Fig. 2). Due to the complex structure, the center of gravity position of the torso + tail must be measured and thus the code largely functions to estimate the associated moment of inertia for the torso. In the study, we performed a sensitivity analysis for up to 15% error of the total torso length on the CG measurement.

1. Calculate the volume of each component assuming an elliptical cylinder for the back piece.
2. Option 1: If the calculated average density places the CG within 5% of the measured value, use the elliptical cylinder and continue to step 5.
3. Option 2: If not, calculate if the average density using an elliptical cone for a back piece places the CG within 5% of the measured value. If so, use the elliptical cone and continue to step 5. Figure 2 illustrates option 2.
4. Option 3: If not, use an elliptical cylinder and an optimization routine to vary the density between each of the three sections of the torso. The routine minimizes the difference between the output densities and the calculated average density for the full torso. In addition, we assume that the CG is moved forwards by 5% of the total length. This is necessary to ensure reasonable densities. We assumed that density measurements could be very low due to the possibility that the majority of the volume is made from loosely packed feathers. The lowest density was found to be 42 kg-m^{-3} for the end section of a storm petrel. All section densities can be seen within the *"VerificationData.xlsx"* tab *"TorsoDensities"* that is included within the publicly available data repository.
5. One of the three above options will provide the final calculated volume, mass and output CG.
6. Given these parameters for each section, calculate $\boldsymbol{I}$ (eqn. 20, 17 and 15) and CG (eqn. 23 and 22) for each component in the torso frame, where $z_{torso} = -x_{fullbird}$, $x_{torso} = z_{fullbird}$ and $y_{torso} = y_{fullbird}$.
7. Transform $\boldsymbol{I}$ and CG to be within the full bird frame and shift so that the VRP is the origin.

5.2.1 Required measurements

1. Mass of the torso and legs, m_{torso}
2. Full torso length, l_{torso}
3. Mass of both legs, m_{leg}
4. Body width at leg insertion, w_{leg}
5. x-location of leg insertion, l_{leg}
6. Maximum body width, w_{max}
7. Maximum body height, h_{max}
8. x-location of maximum body width, l_{bmax}
9. x-location of the CG of torso + legs, CG_x
10. z-location of the CG of torso + legs, CG_z

5.2.2 Assumptions

1. **Minimum density** allowed by the optimizer for the front section is 200 kg/m^3.

Appendix A: Base moment of inertia tensors

All tensors are listed in a published handbook[32] except for the elliptical cone calculation which was computed for this project.

1. **Hollow cylinder.** r_o is the outer radius, r_i is the inner radius, l is the length and m is the mass. Origin is at the center of mass. Used for the bone interior, neck and feather calamus.

$$\boldsymbol{I} = m \begin{bmatrix} \frac{1}{12}(3(r_o^2+r_i^2)+l^2) & 0 & 0 \\ 0 & \frac{1}{12}(3(r_o^2+r_i^2)+l^2) & 0 \\ 0 & 0 & \frac{1}{2}(r_o^2+r_i^2) \end{bmatrix} \tag{13}$$

2. **Solid cylinder.** r is the radius, l is the length and m is the mass. Origin is at the center of mass. Used for the bone end caps and muscles.

$$\boldsymbol{I} = m \begin{bmatrix} \frac{1}{12}(3r^2+l^2) & 0 & 0 \\ 0 & \frac{1}{12}(3r^2+l^2) & 0 \\ 0 & 0 & \frac{1}{2}r^2 \end{bmatrix} \tag{14}$$

3. **Elliptical cylinder.** a is half the maximum height along the x direction, b is half the maximum width along the y direction, l is the length, and m is the mass. Origin is at the center of mass. Used for the last portion of the body if required.

$$I = m \begin{bmatrix} \frac{1}{12}(3b^2+l^2) & 0 & 0 \\ 0 & \frac{1}{12}(3a^2+l^2) & 0 \\ 0 & 0 & \frac{1}{4}(a^2+b^2) \end{bmatrix} \tag{15}$$

4. **Solid cone.** r is the radius of the cone base, h is the height and m is the mass. Origin is at the center of the cone's base, not the center of mass. Used for the head/beak.

$$\boldsymbol{I} = m \begin{bmatrix} \frac{1}{10}(\frac{3}{2}r^2+l^2) & 0 & 0 \\ 0 & \frac{1}{10}(\frac{3}{2}r^2+l^2) & 0 \\ 0 & 0 & \frac{3}{10}r^2 \end{bmatrix} \tag{16}$$

5. **Elliptical cone.** l length until the tip of the cone, A is half the maximum height along the x direction, B is half the maximum width along the y direction, and m is the mass. Origin is at the base of the cone, not the center of mass. Used for the back two-thirds of the body.

$$I = m \begin{bmatrix} \frac{1}{10}(\frac{3}{2}B^2+l^2) & 0 & 0 \\ 0 & \frac{1}{10}(\frac{3}{2}A^2+l^2) & 0 \\ 0 & 0 & \frac{3}{20}(A^2+B^2) \end{bmatrix} \tag{17}$$

6. **Solid square pyramid.** w is the entire width of one side of the pyramid base, h entire height of the pyramid and m is the mass. Origin is at the center of the pyramid's base, not the center of mass. Used for the rachis.

$$\boldsymbol{I} = m \begin{bmatrix} \frac{1}{20}(w^2+2h^2) & 0 & 0 \\ 0 & \frac{1}{20}(w^2+2h^2) & 0 \\ 0 & 0 & \frac{1}{10}w^2 \end{bmatrix} \tag{18}$$

7. **Flat rectangular plate.** w is the entire width of one side, h entire height and m is the mass. Origin is at the center of mass. Used for the feather vanes and tail.

$$\boldsymbol{I} = \frac{1}{12}m \begin{bmatrix} (w^2+h^2) & 0 & 0 \\ 0 & h^2 & 0 \\ 0 & 0 & w^2 \end{bmatrix} \tag{19}$$

8. **Solid hemi-ellipsoid.** a is half the height along the x direction, b is half the width along the y direction and c is half the length along the z direction. Origin is at the base of the hemi-ellipsoid, not the center of mass. Used for the front third of the body.

$$\boldsymbol{I} = \frac{1}{5}m \begin{bmatrix} (b^2+c^2) & 0 & 0 \\ 0 & (a^2+c^2) & 0 \\ 0 & 0 & (a^2+b^2) \end{bmatrix} \tag{20}$$

Appendix B: Base center of gravity vectors

1. **Cylinder.** (hollow, solid, elliptical or circular) l is the length. Origin at the center of the base.

$$CG = \begin{bmatrix} 0 \\ 0 \\ \frac{1}{2}l \end{bmatrix} \tag{21}$$

2. **Pyramid.** (circular, square or elliptical) h is the height. Origin at the center of the base.

$$CG = \begin{bmatrix} 0 \\ 0 \\ \frac{1}{4}h \end{bmatrix} \tag{22}$$

3. **Hemi-ellipsoid.** c is the height. Origin at the center of the base.

$$CG = \begin{bmatrix} 0 \\ 0 \\ \frac{3}{8}c \end{bmatrix} \tag{23}$$

Appendix C: Illustrations of the required measurements

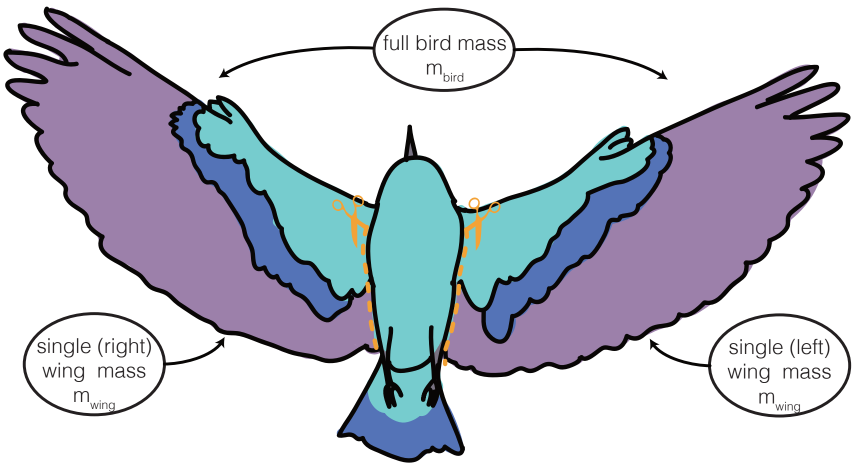

Supplemental Fig. 5 | Entire bird measurements.

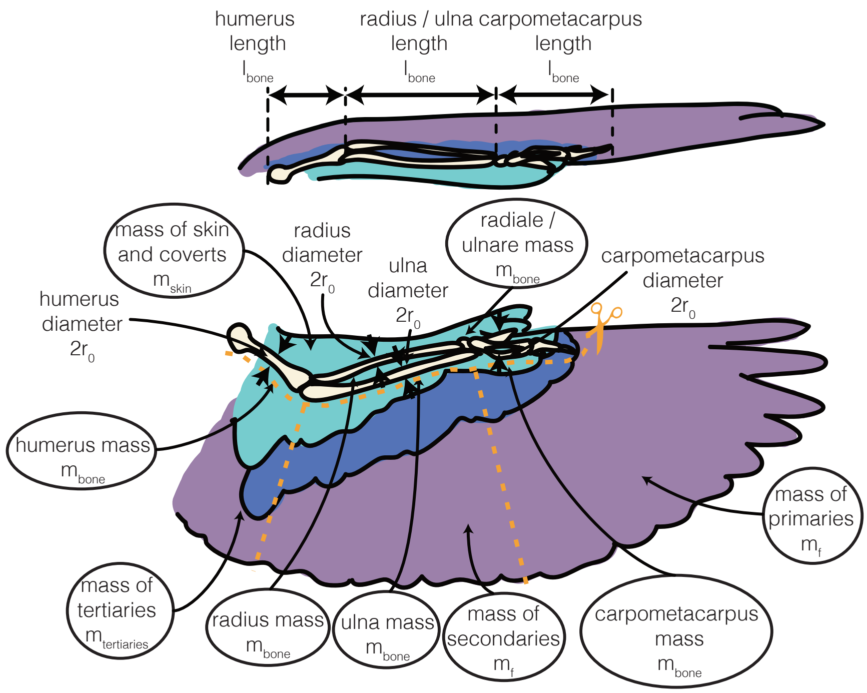

Supplemental Fig. 6 | Wing specific measurements.

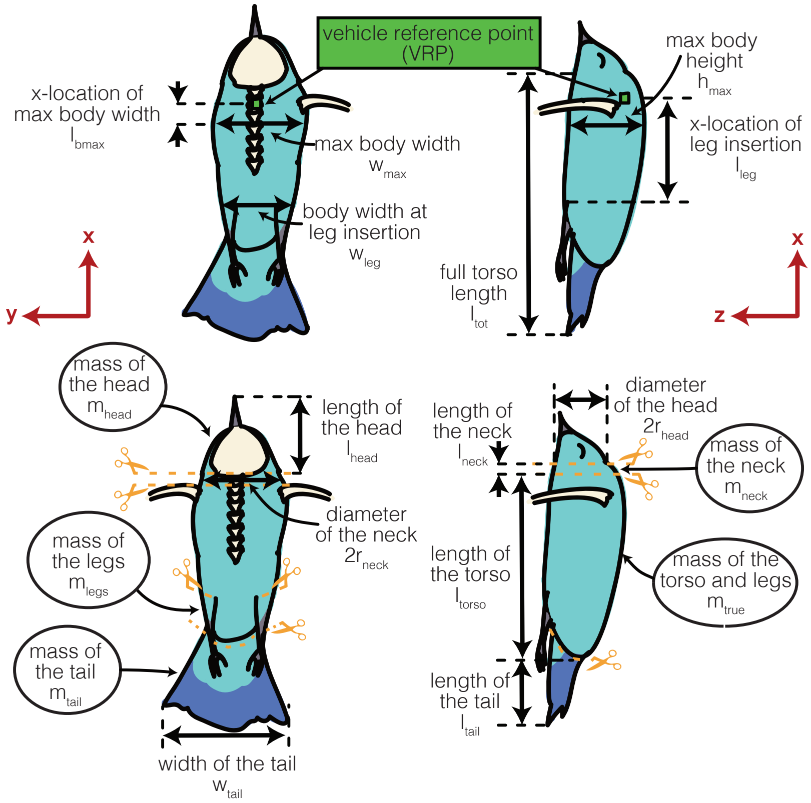

Supplemental Fig. 7 | Body specific measurements.

References

1. Harvey, C., Baliga, V. B., Goates, C. D., Hunsaker, D. F. & Inman, D. J. Gull-inspired joint-driven wing morphing allows adaptive longitudinal flight control. *J. The Royal Soc. Interface* **18**, 20210132, DOI: 10.1098/rsif.2021.0132 (2021). Publisher: Royal Society.
2. Anderson Jr, J. D. *Introduction to Flight* (McGraw-Hill Higher Education, 1989).
3. Pamadi, B. N. *Performance, Stability, Dynamics, and Control of Airplanes* (American Institute of Aeronautics & Astronautics, 2004).
4. Maybury, W., Rayner, J. & Couldrick, L. Lift generation by the avian tail. *Proc. Royal Soc. London. Ser. B: Biol. Sci.* **268**, 1443–1448 (2001).
5. Anderson Jr, J. D. *Fundamentals of aerodynamics* (Tata McGraw-Hill Education, 2010).
6. Liu, T., Kuykendoll, K., Rhew, R. & Jones, S. Avian Wing Geometry and Kinematics. *AIAA J.* **44**, 954–963, DOI: 10.2514/1.16224 (2006).
7. Phillips, W., Hunsaker, D. F. & Niewoehner, R. Estimating the subsonic aerodynamic center and moment components for swept wings. *J. aircraft* **45**, 1033–1043 (2008).
8. Yates, A. H. Notes on the Mean Aerodynamic Chord and the Mean Aerodynamic Centre of a Wing. *The J. Royal Aeronaut. Soc.* **56**, 461–474, DOI: 10.1017/S0368393100129311 (1952). Edition: 2016/07/28 Publisher: Cambridge University Press.
9. Diehl, W. S. The mean aerodynamic chord and the aerodynamic center of a tapered wing. In *Annual Report - National Advisory Committee for Aeronautics, Issues 727-751*, vol. 268 (US Government Printing Office, 1942).
10. Raymer, D. P. *Aircraft Design: A Conceptual Approach* (American Institute of Aeronautics and Astronautics, 1999).
11. Rivera Parga, J. R. *Wind Tunnel Investigation of the Static Stability and Control Effectiveness of a Rotary Tail in a Portable UAV*. Master's thesis, Air Force Institute of Technology, Wright-Patterson Air Force Base, Ohio (2004).
12. Usherwood, J. R. *et al.* High aerodynamic lift from the tail reduces drag in gliding raptors. *The J. Exp. Biol.* **223**, jeb214809, DOI: 10.1242/jeb.214809 (2020).
13. Baruh, H. *Analytical dynamics* (WCB/McGraw-Hill Boston, 1999).
14. Pennycuick, C. The strength of the pigeon's wing bones in relation to their function. *J. Exp. Biol.* **46**, 219–233 (1967). Publisher: The Company of Biologists Ltd.
15. Marelli, C. A. & Simons, E. L. R. Microstructure and Cross-Sectional Shape of Limb Bones in Great Horned Owls and Red-Tailed Hawks: How Do These Features Relate to Differences in Flight and Hunting Behavior? *PLOS ONE* **9**, e106094, DOI: 10.1371/journal.pone.0106094 (2014). Publisher: Public Library of Science.
16. Dumont, E. R. Bone density and the lightweight skeletons of birds. *Proc. Royal Soc. B: Biol. Sci.* **277**, 2193–2198, DOI: 10.1098/rspb.2010.0117 (2010).
17. De Margerie, E., Sanchez, S., Cubo, J. & Castanet, J. Torsional resistance as a principal component of the structural design of long bones: comparative multivariate evidence in birds. *The Anat. Rec. Part A: Discov. Mol. Cell. Evol. Biol. An Off. Publ. Am. Assoc. Anat.* **282**, 49–66, DOI: 10.1002/ar.a.20141 (2005).
18. Bennett, M. & Stafford, J. A. Tensile properties of calcified and uncalcified avian tendons. *J. Zool.* **214**, 343–351, DOI: 10.1111/j.1469-7998.1988.tb04727.x (1988).
19. Hutchinson, J. R. *et al.* Musculoskeletal modelling of an ostrich (Struthio camelus) pelvic limb: influence of limb orientation on muscular capacity during locomotion. *PeerJ* **3**, e1001, DOI: 10.7717/peerj.1001 (2015). Publisher: PeerJ Inc.
20. Bachmann, T., Emmerlich, J., Baumgartner, W., Schneider, J. M. & Wagner, H. Flexural stiffness of feather shafts: geometry rules over material properties. *J. Exp. Biol.* **215**, 405–415, DOI: 10.1242/jeb.059451 (2012).
21. Weiss, I. M. & Kirchner, H. O. The peacock's train (Pavo cristatus and Pavo cristatus mut. alba) I. structure, mechanics, and chemistry of the tail feather coverts. *J. Exp. Zool. Part A: Ecol. Genet. Physiol.* **313**, 690–703, DOI: 10.1002/jez.641 (2010).
22. Pap, P. L. *et al.* Interspecific variation in the structural properties of flight feathers in birds indicates adaptation to flight requirements and habitat. *Funct. Ecol.* **29**, 746–757, DOI: 10.1111/1365-2435.12419 (2015).

23. Rijke, A. M. & Jesser, W. A. The water penetration and repellency of feathers revisited. *The Condor* **113**, 245–254, DOI: cond.2011.100113 (2011). Publisher: Oxford University Press.

24. Filshie, B. & Rogers, G. An electron microscope study of the fine structure of feather keratin. *The J. Cell Biol.* **13**, 1–12, DOI: 10.1083/jcb.13.1.1 (1962). Publisher: Rockefeller University Press.

25. Hertel, H. *Structure, form, movement* (Reinhold Publishing Co., 1966).

26. Wang, B. & Meyers, M. A. Seagull feather shaft: Correlation between structure and mechanical response. *Acta Biomater.* **48**, 270–288, DOI: 10.1016/j.actbio.2016.11.006 (2017).

27. Fraser, R. & MacRae, T. Part II: The Densities of Native Keratins. *Textile Res. J.* **27**, 384–390, DOI: 10.1177/004051755702700507 (1957). Publisher: SAGE Publications Ltd STM.

28. Sullivan, T. N., Wang, B., Espinosa, H. D. & Meyers, M. A. Extreme lightweight structures: avian feathers and bones. *Mater. Today* DOI: 10.1016/j.mattod.2017.02.004 (2017).

29. Elowson, A. Spread-wing postures and the water repellency of feathers: a test of Rijke's hypothesis. *The auk* **101**, 371–383 (1984). Publisher: Oxford University Press.

30. Hally, D. Calculation of the Moments of Polygons. Tech. Rep. AD-A183 444, Defence Research Establishment Suffield Ralston, Alberta, CA (1987).

31. Heers, A. M., Rankin, J. W. & Hutchinson, J. R. Building a Bird: Musculoskeletal Modeling and Simulation of Wing-Assisted Incline Running During Avian Ontogeny. *Front. Bioeng. Biotechnol.* **6**, 140, DOI: 10.3389/fbioe.2018.00140 (2018).

32. Myers, J. A. Handbook of equations for mass and area properties of various geometrical shapes. Tech. Rep., Naval Ordnance Test Station, China Lake, CA, USA (1962).
