# Supplementary material for: Birds can transition between stable and unstable states via wing morphing
Source: Nature. 2022 Mar 9;603(7902):648–53. doi: 10.1038/s41586-022-04477-8 (PMC8942853; doi:10.1038/s41586-022-04477-8)
Supplement: Supplementary file 2 — Reporting Summary [file 41586_2022_4477_MOESM2_ESM.pdf]

nature portfolio

Corresponding author(s): Christina Harvey
Last updated by author(s): Jan 19, 2022

Reporting Summary

Nature Portfolio wishes to improve the reproducibility of the work that we publish. This form provides structure for consistency and transparency in reporting. For further information on Nature Portfolio policies, see our Editorial Policies and the Editorial Policy Checklist.

Statistics

For all statistical analyses, confirm that the following items are present in the figure legend, table legend, main text, or Methods section.

n/a	Confirmed	
☐	☒	The exact sample size (n) for each experimental group/condition, given as a discrete number and unit of measurement
☐	☒	A statement on whether measurements were taken from distinct samples or whether the same sample was measured repeatedly
☐	☒	The statistical test(s) used AND whether they are one- or two-sided<br>*Only common tests should be described solely by name; describe more complex techniques in the Methods section.*
☐	☒	A description of all covariates tested
☐	☒	A description of any assumptions or corrections, such as tests of normality and adjustment for multiple comparisons
☐	☒	A full description of the statistical parameters including central tendency (e.g. means) or other basic estimates (e.g. regression coefficient) AND variation (e.g. standard deviation) or associated estimates of uncertainty (e.g. confidence intervals)
☐	☒	For null hypothesis testing, the test statistic (e.g. F, t, r) with confidence intervals, effect sizes, degrees of freedom and P value noted<br>*Give P values as exact values whenever suitable.*
☐	☒	For Bayesian analysis, information on the choice of priors and Markov chain Monte Carlo settings
☒	☐	For hierarchical and complex designs, identification of the appropriate level for tests and full reporting of outcomes
☐	☒	Estimates of effect sizes (e.g. Cohen's d, Pearson's r), indicating how they were calculated

Our web collection on statistics for biologists contains articles on many of the points above.

Software and code

Policy information about availability of computer code

Data collection	Data collection for the wing range of motion data used Optitrack Motive (Version 2.3.0, https://optitrack.com/support/downloads/motive.html). Data collection for computing inertial properties was completed using a custom R package (AvInertia, Version 0.0.1) that has been released on CRAN (https://cran.r-project.org/web/packages/AvInertia/index.html). All relevant files can be accessed publicly at this time from the Github repository: https://github.com/charvey23/AvInertia.
Data analysis	All data analysis was completed within RStudio (Version 1.3.1093) running R (Version 4.0.3). Feather measurements were extracted using ImageJ (version 2.0.0-rc-43/1.52n).<br><br>The analysis functions can be accessed through the figshare repository (https://doi.org/10.6084/m9.figshare.c.5503989) and the following Github repository: https://github.com/charvey23/AvInertia/tree/master/AnalysisFunctions.<br><br>AvInertia has been publicly released on GitHub (https://github.com/charvey23/AvInertia) and as a CRAN package (https://cran.r-project.org/package=AvInertia).

For manuscripts utilizing custom algorithms or software that are central to the research but not yet described in published literature, software must be made available to editors and reviewers. We strongly encourage code deposition in a community repository (e.g. GitHub). See the Nature Portfolio guidelines for submitting code & software for further information.

Data

Policy information about availability of data

All manuscripts must include a data availability statement. This statement should provide the following information, where applicable:

- Accession codes, unique identifiers, or web links for publicly available datasets
- A description of any restrictions on data availability
- For clinical datasets or third party data, please ensure that the statement adheres to our policy

All data reported and used in this paper have been deposited in public repositories identified within: https://doi.org/10.6084/m9.figshare.c.5503989. Relevant files are located within: "Data output files: Birds can transition between stable and unstable states via wing morphing" and "Data input files: Birds can transition between stable and unstable states via wing morphing".

Field-specific reporting

Please select the one below that is the best fit for your research. If you are not sure, read the appropriate sections before making your selection.

☐ Life sciences ☐ Behavioural & social sciences ☒ Ecological, evolutionary & environmental sciences

For a reference copy of the document with all sections, see nature.com/documents/nr-reporting-summary-flat.pdf

Ecological, evolutionary & environmental sciences study design

All studies must disclose on these points even when the disclosure is negative.

Study description

In this study we used morphological measurements from specimens combined with wing range of motion data to calculate the inertial properties of a bird throughout wing morphing. We obtained morphological data for 36 adult specimens representing 22 species (Fig. 2a) from frozen cadavers acquired from the Cowan Tetrapod Collection at the Beaty Biodiversity Museum (University of British Columbia, Vancouver, Canada).

Research sample

We obtained morphological data for 36 adult specimens representing 22 species from frozen cadavers acquired from the Cowan Tetrapod Collection at the Beaty Biodiversity Museum (University of British Columbia, Vancouver, Canada). Sample size was a function of the availability and quality of specimens from the Beaty Biodiversity Museum as we could only rely on fully-intact, well-preserved specimens. We attempted to acquire multiple specimens of the same species when possible. We selected species to span the phylogeny defined by Prum et al. except for Palaeognathae as this clade contains largely flightless birds. In all we had specimens from the following species:

Accipiter cooperii x1 - adult male
Accipiter striatus x1 - adult male
Aechmophorus occidentalis x2- adult unknown
Anas platyrhynchos x2 - adult male
Ardea herodias x2- adult unknown
Branta canadensis x1- adult unknown
Chordeiles minor x2- adult unknown
Chrysolophus amherstiae x1 - adult male
Colaptes auratus x2 - adult male
Columba livia x3- adult unknown
Corvus corax x1- adult unknown
Cyanocitta stelleri x2- adult unknown
Cypseloides niger x1- adult unknown
Falco columbarius x3 - adult male
Falco peregrinus x1 - adult unknown
Larus glaucescens x1 - adult unknown
Lophophorus impejanus x1 - adult male
Lophura nycthemera x1 - adult male
Megaceryle alcyon x3 - adult male
Hydrobates leucorhous x2 - adult unknown
Pelecanus erythrorhynchos x1 - adult unknown
Tyto alba x2 - adult male

The cadavers were inspected to ensure adequate condition and completeness, after which we measured the full body mass, wingspan, and body length. Next, we disarticulated the wing at the shoulder joint, taking care to ensure that each wing's skin, propatagial elements, and feathers remained intact. One wing from each cadaver was used to determine wing range of motion (ROM) and corresponding wing shape change. The cadaver was further dissected to obtain length and mass measurements for the head, neck, torso, wing components, legs, and tail (refer to Supplementary Information for details on each measurement).

To determine the wing ROM and corresponding shape change, we actuated the cadaver wings throughout the full range of extension and flexion of the elbow and wrist joints by hand.

Sampling strategy

Sample size was a function of the availability and quality of specimens from the Beaty Biodiversity Museum as we could only rely on fully-intact, well-preserved specimens. We attempted to acquire multiple specimens of the same species when possible. Further

	details are contained within relevant sections of the manuscript are methods sections "Phylogenetic and statistical analyses" and "Sensitivity analysis".
Data collection	All required morphological measurements and wing range of motion videos were obtained by V.B. Baliga and J.C.M. Wong. Morphological measurements obtained manually on each specimen were recorded in Microsoft Excel 2021. Wing ROM was obtained using four to five cameras and Optitrack Motive (Version 2.3.0, https://optitrack.com/support/downloads/motive.html). Additional details are contained within relevant sections of the manuscript are methods sections "Collection of morphological data " and "Determination of the elbow and wrist range of motion".
Timing and spatial scale	All morphological measurements were obtained between March 2020 and March 2021. There was approximately a 5 month delay in data collection due to COVID-19 from April 2020-September 2020. Timing was a function of the date that the specimen was acquired from the museum and the lab schedule dictated by COVID-19 protocols. Frozen specimens were fully thawed before measurements were collected and all cadavers were subsequently disposed of due to the destructive nature of the measurements. All wing range of motion measurements took approximately 20 min.
Data exclusions	No data was excluded from this study.
Reproducibility	For multiple species (11/22) we acquired more than one specimen which allowed us to calculate and display the results for each individual. Refer to "Research Sample" section above for the number of replicates per species. All replicates were included within the analysis and are included within data figures. We observed minor variation between results of a species attributable to expected individual specific variation.
Randomization	This study consisted of a single experimental group and thus randomization was not necessary.
Blinding	This study consisted of a single experimental group and thus blinding was not necessary.

Did the study involve field work? ☐ Yes ☒ No

Reporting for specific materials, systems and methods

We require information from authors about some types of materials, experimental systems and methods used in many studies. Here, indicate whether each material, system or method listed is relevant to your study. If you are not sure if a list item applies to your research, read the appropriate section before selecting a response.

Materials & experimental systems

n/a	Involved in the study
☒	☐ Antibodies
☒	☐ Eukaryotic cell lines
☒	☐ Palaeontology and archaeology
☐	☒ Animals and other organisms
☒	☐ Human research participants
☒	☐ Clinical data
☒	☐ Dual use research of concern

Methods

n/a	Involved in the study
☒	☐ ChIP-seq
☒	☐ Flow cytometry
☒	☐ MRI-based neuroimaging

Animals and other organisms

Policy information about studies involving animals; ARRIVE guidelines recommended for reporting animal research

Laboratory animals	This study did not involve laboratory animals.
Wild animals	We obtained morphological data for 36 adult specimens representing 22 species from frozen cadavers acquired from the Cowan Tetrapod Collection at the Beaty Biodiversity Museum (University of British Columbia, Vancouver, Canada).
Field-collected samples	This study did not involve samples collected from the field. All of the specimen used were cadavers obtained from the muesum.
Ethics oversight	No ethical approval or guidance was required for this work. Museum acquired these cadavers following the necessary guidelines and with a wildlife act permit (SU16-236177). Cadavers were used for wing ROM study and morphological measurements and subsequently disposed following appropriate biological waste requirements.

Note that full information on the approval of the study protocol must also be provided in the manuscript.
