# Supplementary material for: Birds can transition between stable and unstable states via wing morphing
Source: Nature. 2022 Mar 9;603(7902):648–53. doi: 10.1038/s41586-022-04477-8 (PMC8942853; doi:10.1038/s41586-022-04477-8)
Supplement: Supplementary file 3 — Peer Review File [file 41586_2022_4477_MOESM3_ESM.pdf]

Peer Review File

Manuscript Title: Birds can transition between stable and unstable states via wing morphing

Reviewer Comments & Author Rebuttals

Reviewer Reports on the Initial Version:

Referee #1 (Remarks to the Author):

This is an exciting and innovative study that draws insight from avian anatomy, recent excellent papers (largely by the same authors), phylogenetic method in biology, and classical aeronautical literature, to address a major question regarding the evolution of agility in birds. The manuscript is well written, well explained, and shows interesting findings about how phylogenetic signal in the range of motion at the wrist and elbow contribute to potential mode switching between stable and unstable configurations in birds of a range of sizes. This is accomplished using a new method for assessing moments of inertia and centres of gravity, and using the results of that method in conjunction with justifiable assumptions about the aerodynamic centre of the wings and the neutral point of the avian airframe.

There is no doubt that this work addresses a gap in our understanding, or at least an area where our current understanding lags behind other elements of the full picture that we are yet to see.

The methods are solid and give a robust result within the parameters and limits that have been set.

The authors acknowledge that they are taking a step towards a full synthesis, rather than reaching that point with this work. This is an impressive piece of work but ultimately rather limited. Lines 188 to 191 are an accurate summary. It is a crucial step in the right direction but the brush may be too broad to be the full story we need to understand avian agility.

Specifically (paragraph ~Line 100): I fear that without shoulder motion this is this is missing substantial morphing that will lead to more significant changes in the centre of gravity. Shoulder movement rotates the whole wing (importantly, including the heavier, proximal portions) and also shifts the neutral point much further than the elbow and wrist motion. Fig 2b appears to show that shoulder motion dominates. I’m surprised, then, that shoulder rotation is not a more prominent feature of the analysis in the paper when it is likely to be most important for pitch control (and possibly other rotations about intermediate axes). I recommend strengthening this element.

The challenge is that including rotations of the shoulder are likely to give both stable and unstable results. This could be interpreted as birds maintaining the ability to transition between stable and unstable configurations, but it would remain inconclusive how it was done, or if both configurations were used in flight. There may also, in vivo, be strong correlations between the shoulder articulation and articulation at the elbow and wrist.

Specific comments:

Abstract: Given the geometric relationship between mass and length, I found this sentence in the abstract to be worded rather confusingly: “We found that absolute agility scales with body mass but once normalized by body length and flight speed, pitch agility capacity is independent of body mass.”

I agree with the authors that there is a dearth of information on mass distribution and hence the inertial properties of birds. However, there is, perhaps, a longer history of consideration of bird centres of gravity and moment of inertia than the authors currently credit.

e.g. (but not limited to):

https://journals.biologists.com/jeb/article/222/9/jeb185488/682/Avian-surface-reconstruction-in-free-flight-with

or

https://link.springer.com/article/10.1007/s00332-021-09698-1

Relevant analyses can also be found for bats, microraptor, and so on, as well as studies of birds where the centre of mass of the wings and body have been considered separately. Using the range of movement data, this study goes far further than past work, but it is not quite true to say that it is not known *if* the CoG moves (L56). It must; and we know this both by a combination of observation and logical deduction (wings move up and down) and because it has been measured/estimated previously.

Referee #2 (Remarks to the Author):

The paper quantifies the inertial properties of 22 bird species. The authors state that deriving the equations of motion for birds to assess maneuverability and stability would not be possible without such inertial measurements and calculations. I believe that this paper and these results make a

contribution towards understanding and assessing birds' flight. I have two major concerns and a few comments.

Major Concerns:

1. The assumption that the wing aerodynamic center and neutral point are collocated at the quarter chord of the wing root is an oversimplification. Since the authors have the planform shape of the wings, they can easily calculate the mean chord and assume that the wing aerodynamic center is at the quarter chord of the mean chord, not the root chord. Using the c/4 of the mean chord would better capture the fact that the aerodynamic center of the wing could vary as a function of the wrist and elbow angle, especially at the limits of the ROM. Second, while the tail is furled, for birds with relatively long body length, the tail may shift the neutral point. I think this study could benefit from testing the inertial findings assuming different locations for the neutral point (i.e., would the paper's conclusions hold if the authors accounted for the aerodynamic tails?)

2. Did the authors characterize the effect of the CG shift due to the wrist and elbow flexion as a function of the arm to hand wing ratio? Are the species tested represent a group of species where the hand wing is significantly smaller than the arm wing, and would the results change if the species tested were broader?

Other Comments

• I (the moment of inertia) is a function of time and flight configuration. What are the time scales for wrist and elbow extension during flying? Have the authors considered recommending a parametric oscillation model for the equations of motion? Such a model could affect the stability and maneuverability metrics

• Can the authors justify the statement in lines 109-110?

• Please label the view for Figure 1 e.

• It is hard to understand the ROM from Figure 1g. What do the square brackets mean? Is the ROM normalized by the wingspan? Would you please clarify figure 1g

• The manuscript would benefit from a strong summary paragraph that highlights the main takeaways of the article.

Referee #3 (Remarks to the Author):

A. Summary of the key results.

This manuscript explores the effects of wing morphing on the mass distribution of birds, and the consequent effects on stability and control. It does so by measuring the range of motion inherent in the wings of cadavers across a range of taxa, which it uses to inform modelling of the changes in centre of mass (CoM) and inertia tensor (I). The authors show that a realistic range of wrist and elbow motion has comparatively small effects on the longitudinal position of the CoM, but that shoulder flexion can affect CoM position substantially. Conversely, a realistic range of wrist and elbow flexion has large effects on the roll and yaw moment of inertia, but little effect on the pitch moment of inertia. A phylogenetically controlled analysis is used to analyse the evolution of maximally and minimally stable configurations in birds under an Ornstein Uhlenbeck model, leading the authors to conclude that birds have evolved to be able to switch between stable and unstable flight configurations. This is contrasted with the orthodoxy first introduced by Maynard Smith (1952), that birds became increasingly unstable through evolution.

B. Originality and significance

As the authors note, most research on avian flight stability focusses on aerodynamics, although the importance of mass distribution is well known, and was analysed for cadavers with fully spread wings (i.e. without morphing) by Thomas and Taylor (2001) in Ref. 12. That paper concluded “it seems reasonable to suggest that an animal that is statically stable during normal flight should not deliberately make itself unstable, except perhaps transiently during unsteady manoeuvres. … If it is found that flying animals do switch facultatively between stable and unstable flight, then this will surely represent one of the most remarkable capabilities of their locomotor control systems.” That being so, the present paper’s key conclusion that “evolutionarily pressures may be maintaining the ability to shift between stable and unstable configurations” is indeed a significant one – particularly given current interest in morphing-wing vehicles. I would encourage the authors to review the concluding paragraph of Ref 12, and particularly the text replaced by the ellipsis above, to see if they agree that there are implications for control reversal that may be worthy of further discussion. (Perhaps not during unsteady manoeuvres if morphing is used only transiently, but presumably so in steady manoeuvres if morphing is used to switch between stable versus unstable setpoints.)

C. Data & methodology: validity of approach, quality of data, quality of presentation

The overall approach is appropriate to answering questions on the effects of varying mass distribution, but it seems a little surprising that the authors have not attempted to overcome the main limitation of their study that they identify (lines 183-5). Specifically, the stability metrics that they compute are based on the position of the CoM relative to the wing’s aerodynamic centre (AC), but only take account of variation in the former. It is possible I have misunderstood the Methods on this point, but as the authors assume that the aerodynamic centre of the wing is located at its quarter-chord point, it would presumably be possible to model its movement with changes in wing position, based on the information they have captured on wing motion. (Note: line 152 refers this

position to one quarter of the root chord, but this is unlikely to be accurate for a highly swept wing, for which I would have assumed that the mean chord would be the more accurate measure.) In principle, the main conclusion of the paper, that “evolutionarily pressures may be maintaining the ability to shift between stable and unstable configurations” would be stronger if the metric upon which this were based had been calculated to include the coupled motion of the CoM and AC during wing morphing. That said, the limitation is noted appropriately at the point that this conclusion is made.

The quality of the data is excellent: the species are well-chosen for comparative analysis, and the estimates of mass distribution appear to be well done, supported by a detailed description of the R package that the authors created to implement this. The graphical presentation of the figures is generally excellent, but symbols and abbreviations are not always explained in the legends and the very brief form of the legends makes it impossible to be certain what is being shown in some panels. I appreciate that Nature constrains figure legend lengths for papers with Methods sections, but in that case, the detail needs to be unambiguously provided in the Methods.

D. Appropriate use of statistics and treatment of uncertainties

The use of statistics and treatment of uncertainties seems broadly appropriate, but the description of the model of trait evolution and the legends and axis labels for the accompanying figures seem somewhat incomplete. I would recommend considering review of this section by a specialist statistician, given that there are certain difficulties involved in the interpretation of these models – particularly at small sample sizes (see: Natalie Cooper, Gavin H. Thomas, Chris Venditti, Andrew Meade, Rob P. Freckleton, A cautionary note on the use of Ornstein Uhlenbeck models in macroevolutionary studies, Biological Journal of the Linnean Society, Volume 118, Issue 1, May 2016, Pages 64–77, https://doi.org/10.1111/bij.12701).

Currently, the authors' main conclusions seem to be relying quite heavily on model estimates of what appear to be weak but robust selection pressures. It would therefore be reassuring to report more fully on the actual measured distribution of the maximum and minimum stability ratios. I think this may be what is shown in Fig. 4e, but attractive as this panel is, it is not entirely clear whether it is supposed to show the results of measurements or the model, and it would be useful to support this with a basic plot of the data.

Ultimately, unless a large majority of the species are shown empirically to have a range of CoM motion that allows them to transition between stable and unstable configurations, the main conclusion of the manuscript is heavily dependent on the results of a somewhat opaque model of evolution. I’d therefore encourage adding some basic statistics on the median stability/instability of the maximum and minimum configurations, which are relevant anyway to understanding how general the authors' findings may be across extant birds.

I am not sure whether I am reading Extended Data Figure 1 correctly, which reflects the fact that the legend needs some improvement. However, on the face of it, this figure seems to show a bias in the Brownian model (which seems rather surprising), and an extremely small difference between the observed maximum and minimum stability ratios in comparison to the range of variation in the Monte Carlo analysis (much smaller than the Results seem to report). I therefore reserve judgement on this analysis pending fuller description in a revised Methods and Results section, and fuller elaboration of the figure legends, etc.

E. Conclusions: robustness, validity, reliability

The key limitation of this paper is that the method appears to take account only of the movement of the CoM as the wings flex, and not of coupled movement of the AC. This seems a surprising omission given the authors' statement (line 78) that their own "previous studies have shown that this range of motion (ROM) allows a substantial shift in the neutral point" (where the neutral point is equated with the AC). In fact, I am left wondering whether this reflects an omission in the description of the Methods, rather than an omission in the analysis, and suggest that the authors revise lines 268-271 for clarity. That said, it seems unlikely that taking account of coupled AC motion would fundamentally alter the conclusions, because the AC and CoM are likely to move fore and aft together. Even so, the conclusions would be much more robust if this were analysed.

I am also not entirely convinced by the conclusion "Our results indicate that it is unlikely that flight dynamics during elbow and wrist flexion and extension will be substantially affected by a shifting CG." This conclusion seems to be based on the result that the CoM shifts by a maximum of 3% of body length under realistic flexion of the wrist and elbow. However, as the rest of the paper shows, the length of the body is not the relevant measure, because (i) statically stable systems become statically unstable in a threshold manner; and (ii) what therefore matters is the movement in relation to the static margin (i.e. the difference between the position of the CoM and AC as a fraction of the chord). I'd suggest restating the results accordingly, with appropriate normalisation.

It is also not clear to me how the conclusion that CoM movement during elbow/wrist flexion has little effect on flight dynamics relates to the authors' main conclusion that birds have evolved to sit at the threshold of stability versus instability. Presumably, this is because the maximum versus minimum stability measures take account of either the much larger effect of shoulder motion, or a wider range of elbow/wrist motion? If so, this needs clarification in the text, as the definition of the maximum and minimum stability metrics does not appear to be clearly stated in the text.

F. Suggested improvements: experiments, data for possible revision

Besides the major comments above, I would suggest the following detailed revisions:

(i) The main text at line 167 and following refers to a “log-transformed static pitch stability ratio”, which I assume is the same as the “log transformed static margin ratio” defined at Eq. 7. Please make the terminology consistent, and if using the “static margin” terminology, then note that this jargon is not defined in the paper.

(ii) There is no logarithm shown in Eq. 7, which makes the meaning of “log-transformed” and the data to which this refers rather ambiguous. I assume the authors mean that they log-transformed Eq. 7. If so, they should show this, and confirm the base of the logarithm, which is needed for the numerical results of the OU model to be interpretable.

(iii) The Methods need further clarification throughout, particularly in relation to the calculation of the position of the AC.

(iv) The definitions of manoeuvrability (line 26) and especially agility (line 64) are stated rather imprecisely, and I would struggle to quantify either without further explanation.

(v) It would help to use bold font to show that Eqs. 1-2 are matrix equations, particularly having said that there are 6 of these equations.

(vi) The definition of static stability at line 66 as “the inherent tendency to return to an initial equilibrium after a disturbance” reads more like a definition of dynamic stability. I would say “the initial tendency to return towards equilibrium after a disturbance”.

(vii) At line 73 “across all avian clades” rather neglects the fact that a clade can be defined at any level within a phylogeny. I know what the authors are trying to say, but clade needs some qualification here in terms of taxonomic level.

(viii) Lines 85-97 describe validation of the method against old data. Whilst useful, I doubt that most readers will be interested in the detail, and suggest moving this to Methods.

(ix) Line 93 “encapsulated” should probably be “encompassed”, or the sentence could be restructured to describe this in terms of intervals.

(x) At line 158 and in Fig. 4 “agility capacity” is used as if this is a precise quantity, but the meaning of the term is nowhere defined. I assume this is the “pitch agility metric” defined at Eq. 3 in the Methods, but again, the terminology needs to be consistent.

(xi) Some figure legends need expansion to make the figures understandable, and some axes are missing labels or units (e.g. Extended Data Figure 1).

(xii) At Line 269, I can’t parse the phrase “the neutral point is proportional to the wing’s theoretical aerodynamic centre”.

(xiii) It is not completely clear how the maximum and minimum stability ratios are defined. There needs to be a clear statement somewhere of the wing configurations to which these refer, in terms of which joints and flexing, and through what angle(s).

F. References: appropriate credit to previous work?

Suggest considering the following:

Hedrick TL, Usherwood JR, Biewener AA. Wing inertia and whole-body acceleration: an analysis of instantaneous aerodynamic force production in cockatiels (Nymphicus hollandicus) flying across a range of speeds. J Exp Biol. 2004 Apr;207(Pt 10):1689-702. doi: 10.1242/jeb.00933. PMID: 15073202. [Detailed measurements of wing inertia.]

Mills, R., Hildenbrandt, H., Taylor, G.K., Hemelrijk, C.K. (2018). Physics-based simulations of aerial attacks by peregrine falcons reveal that stooping at high speed maximizes catch success against agile prey. PLoS Comput. Biol.14(4), e1006044. https://doi.org/10.1371/journal.pcbi.1006044. [Modelling of variation in roll inertia during manoeuvres].

Cheney JA, Stevenson JPJ, Durston NE, Maeda M, Song J, Megson-Smith DA, Windsor SP, Usherwood JR, Bomphrey RJ. Raptor wing morphing with flight speed. J R Soc Interface. 2021 Jul;18(180):20210349. doi: 10.1098/rsif.2021.0349. Epub 2021 Jul 14. PMID: 34255986; PMCID: PMC8277465. [Recent work probably post-dating submission].

G.Clarity and context: lucidity of abstract/summary, appropriateness of abstract, introduction and conclusions.

The writing is mostly clear, but there are some inconsistencies in terminology between the Methods and Main Text (see above), and points where the language would benefit from some tightening up for precision (see above). In places, the text seems aimed at a more specialist audience than the readership of Nature, notably the section on model validation.

Author Rebuttals to Initial Comments:

Referee #1:

This is an exciting and innovative study that draws insight from avian anatomy, recent excellent papers (largely by the same authors), phylogenetic method in biology, and classical aeronautical literature, to address a major question regarding the evolution of agility in birds. The manuscript is well written, well explained, and shows interesting findings about how phylogenetic signal in the range of motion at the wrist and elbow contribute to potential mode switching between stable and unstable configurations in birds of a range of sizes. This is accomplished using a new method for assessing moments of inertia and centres of gravity, and using the results of that method in conjunction with justifiable assumptions about the aerodynamic centre of the wings and the neutral point of the avian airframe.

There is no doubt that this work addresses a gap in our understanding, or at least an area where our current understanding lags behind other elements of the full picture that we are yet to see.

The methods are solid and give a robust result within the parameters and limits that have been set.

Thank you for your supportive comments. We are grateful for all the reviewers' suggestions as we believe that this has led to a substantial improvement of our manuscript. We have addressed each specific concern below in detail.

The authors acknowledge that they are taking a step towards a full synthesis, rather than reaching that point with this work. This is an impressive piece of work but ultimately rather limited. Lines 188 to 191 are an accurate summary. It is a crucial step in the right direction but the brush may be too broad to be the full story we need to understand avian agility.

Specifically (paragraph ~Line 100): I fear that without shoulder motion this is this is missing substantial morphing that will lead to more significant changes in the centre of gravity. Shoulder movement rotates the whole wing (importantly, including the heavier, proximal portions) and also shifts the neutral point much further than the elbow and wrist motion. Fig 2b appears to show that shoulder motion dominates. I'm surprised, then, that shoulder rotation is not a more prominent feature of the analysis in the paper when it is likely to be most important for pitch control (and possibly other rotations about intermediate axes). I recommend strengthening this element.
The challenge is that including rotations of the shoulder are likely to give both stable and unstable results. This could be interpreted as birds maintaining the ability to transition between stable and unstable configurations, but it would remain inconclusive how it was done, or if both configurations were used in flight. There may also, in vivo, be strong correlations between the shoulder articulation and articulation at the elbow and wrist.

We agree that understanding the role of the shoulder joint will be important to continue developing our understanding of avian maneuverability. In this work we limited our scope to solely the elbow and wrist joints for a few key reasons. Primarily, the shoulder joint can be both swept and/or raised to further stabilize or destabilize from the set position used in this study. As such, it is our expectation that shoulder motion will largely serve to enhance the stability or instability available from the elbow and wrist alone. We expect this to be the case because UAV studies have shown that the motion of the neutral point is substantially larger than the motion of the centre of gravity due to wing sweep (Neal et al., 2004, Ref. 25) and that shoulder-inspired joint dihedral provides effective control over the longitudinal axis (Paranjape et al., 2011, Ref 26). Because our results show that the elbow and wrist joints alone allow both stable and unstable configurations, we expect that adding in the shoulder motion will not detract from this result with respect to each species' absolute capacity. We do believe that this expectation should be verified by future studies to define the entire

capacity of bird wing morphing to adjust stability and further move us towards a complete understanding of avian maneuverability. To this end, we have addressed this concern in four locations in the manuscript as follows:

Line 82:
"*In addition, we assumed that the shoulder was set to allow a comparable wing orientation (see methods) and that the tail is furled, but these degrees of freedom play an important role in avian flight control and warrant future morphing studies."*

Line 143:
"Incorporating the shoulder joint range of motion would increase the inertial pitch control provided by the wings."

Line 155:
"*Note that the shoulder joint would broaden the static margin range because the resultant neutral point shift is expected to be larger than the CG shift as evidenced by the control effectiveness of shoulder-joint inspired actuators on morphing UAVs*[25,26]."

Line 193:
"It is important to highlight that further analytical and experimental studies are also necessary to incorporate the variable aerodynamic capabilities of each species, shoulder and tail range of motion and in vivo wing and tail shapes to definitively confirm the optimal phenotype(s) for static pitch stability. Because the shoulder joint will likely serve to increase the static margin range, we expect that this will enhance the ability to shift between stability modes and the available pitch control, although the extent of this enhancement will depend on each species' shoulder ROM[5,25,26]*."*

Additionally, we agree that *in vivo* studies linking the shoulder articulation to elbow and wrist articulation will be an exciting and crucial next step in our understanding of bird flight but this is outside of the scope of our current study. To highlight these important next steps directly, we have both addressed this within Line 193 (see above) and added a new sentence to the manuscript as follows:

Line 186:
"Elbow and wrist flexion and extension alone offers birds the capacity to shift between these pitch stability modes but, if and when a flying bird does shift between these modes remains to be seen."

Specific comments:

Abstract: Given the geometric relationship between mass and length, I found this sentence in the abstract to be worded rather confusingly: "We found that absolute agility scales with body mass but once normalized by body length and flight speed, pitch agility capacity is independent of body mass."

We apologize for the confusing wording. The introduced normalization allows comparison across birds of different sizes and flying at different speeds, similar to that used to compare across aircraft. Due to the newly implemented improvements to the estimation of the neutral point, this result has changed. This change is driven by larger relative static margin shifts than was predicted from the root chord alone. This is expected because the root chord estimation does not allow for the neutral point to move in front of the shoulder joint. For more details on the aerodynamic estimations, we have included a new supplemental methods document entitled "Supplemental methods: Aerodynamic approximations". We have adjusted this abstract sentence to read:

Line 17:

"With the addition of inertial characteristics, we derived a novel metric of pitch agility and estimated the static pitch stability, which revealed that the agility and static margin range are reduced as body mass increases."

Additionally, we have added a sentence in the methods section explaining how the metric was normalized as follows:

Line 323:

"As the units of equation (3) are s^{-2}*, we non-dimensionalized this metric by multiplying by the square of the full bird's length divided by* V^2*. This normalization allows us to compare birds as if they are the same length and flying at the same speed."*

I agree with the authors that there is a dearth of information on mass distribution and hence the inertial properties of birds. However, there is, perhaps, a longer history of consideration of bird centres of gravity and moment of inertia than the authors currently credit.
e.g. (but not limited to):
https://journals.biologists.com/jeb/article/222/9/jeb185488/682/Avian-surface-reconstruction-in-free-flight-with
or
https://link.springer.com/article/10.1007/s00332-021-09698-1

Relevant analyses can also be found for bats, microraptor, and so on, as well as studies of birds where the centre of mass of the wings and body have been considered separately. Using the range of movement data, this study goes far further than past work, but it is not quite true to say that it is not known *if* the CoG moves (L56). It must; and we know this both by a combination of observation and logical deduction (wings move up and down) and because it has been measured/estimated previously.

Thank you for highlighting this concern. We agree that stating "if the CG moves" was an oversimplification and our contribution is related to "how much it moves" with respect to the wing morphing. We have revised the highlighted sentence as follows:

Line 56:

"Physically shifting a bird's morphology will shift the CG but, it is not currently known how much the CG moves as a bird morphs."

We have added these two citations along with other key studies to a revised sentence in the introduction as follows:

Line 48:

"However, studies often overlook the equally essential inertial properties (Fig. 1a) or use static morphology approximations for individual species[13,17–20]."

Referee #2:

The paper quantifies the inertial properties of 22 bird species. The authors state that deriving the equations of motion for birds to assess maneuverability and stability would not be possible without such inertial measurements and calculations. I believe that this paper and these results make a contribution towards understanding and assessing birds' flight. I have two major concerns and a few comments.

Thank you for your comments and suggestions. We believe that we have been able to address your major concerns by adjusting our analysis to account for your suggested changes. We are grateful for all the reviewers' suggestions as we believe that this has led to a substantial improvement of our manuscript.

Major Concerns:

1. The assumption that the wing aerodynamic center and neutral point are collocated at the quarter chord of the wing root is an oversimplification. Since the authors have the planform shape of the wings, they can easily calculate the mean chord and assume that the wing aerodynamic center is at the quarter chord of the mean chord, not the root chord. Using the c/4 of the mean chord would better capture the fact that the aerodynamic center of the wing could vary as a function of the wrist and elbow angle, especially at the limits of the ROM.

Thank you for highlighting this concern, which was also raised by referee #3. We agree that the estimation of the neutral point should be better quantified. To address this concern, we leveraged the results from our previously published study on gull wing-body configurations to investigate which chord metric scaled with the estimated neutral point throughout elbow and wrist flexion and extension. We compared a total of 6 different metrics and found that the quarter chord point of the standard mean chord was the best at approximating the known neutral point for the gull wing-body configurations. We have detailed this analysis in a new supplementary document entitled "Supplemental methods: Aerodynamic approximations". As a result of this investigation, we have adjusted our analysis of stability and agility to incorporate this improved aerodynamically informed metric.

We are grateful that this concern was highlighted as we believe it improves the applicability of our results. As this chord metric was selected solely based on a rigid gull wing study, we have kept an adjusted version of our qualifying statement about how this work must be verified for other species as follows:

Line 193:
"It is important to highlight that further analytical and experimental studies are also necessary to incorporate the variable aerodynamic capabilities of each species, shoulder and tail range of motion and in vivo wing and tail shapes to definitively confirm the optimal phenotype(s) for static pitch stability."

However, we believe that the adjusted analysis with the mean chord metric will now better account for the aerodynamic implications of elbow and wrist morphing across all wings. Of note, this change affected two conclusions about the pitch agility: we found that the normalized agility metric now scales negatively with mass and that there is evidence of selection acting on the upper bound of agility. As agility is largely informed by this static margin, we selected to focus the discussion on evolutionary pressure on the static margin itself. To this end, our revised stability investigation provides evidence supporting the ability to transition between stable and unstable flight albeit with a stronger selection pressure towards stability. This difference was expected because the neutral point predicted with the standard mean chord is predominately more aft than that predicted from the root chord, but as the reviewer highlighted, this does give a more accurate estimation than the root chord. In all, we have created a new supplemental document and substantially revised the final

five paragraphs of the paper as well as the methods section "Agility and stability metrics" to incorporate these changes.

Second, while the tail is furled, for birds with relatively long body length, the tail may shift the neutral point. I think this study could benefit from testing the inertial findings assuming different locations for the neutral point (i.e., would the paper's conclusions hold if the authors accounted for the aerodynamic tails?)

We agree the implication of the tail at a moment arm from the center of gravity is important to estimate. As it is outside of the scope to experimentally determine the aerodynamic characteristics of each species' tail we do not have detailed aerodynamic data that accounts for possible non-linearities in the aerodynamic results. But, there are standard aerodynamic relationships that we used to shed some light on the possibilities. We used a standard aerodynamic model to estimate the shift of the neutral point due to the furled tail and provided full details of this analysis in Section 2 of the new supplementary document entitled "Supplemental methods: Aerodynamic approximations". We found that even when accounting for the tail shift, our results do provide evidence for selection tending to maintain the ability to shift between stable and unstable states although the absolute result is shifted towards the stable range. We adjusted a sentence in the manuscript to highlight this analysis as follows:

Line 174:
"*The strength of selection (α) was relatively low, but our results were robust to measurement errors (Extended Data Fig. 3) and to a preliminary estimation of a neutral point shift due to the tail (electronic supplemental materials).*"

This new analysis accounted for possible shifts due to a furled tail as that is the configuration discussed within our study but to further advance towards a complete understanding of avian agility it will be necessary to understand how tail morphing affects the final results. Within the manuscript, we added more direct statements concerning the need for future studies focused on tail morphing degrees of freedom as follows:

Line 82:
"In addition, we assumed that the shoulder was set to allow a comparable wing orientation (see methods) and that the tail is furled, but these degrees of freedom play an important role in avian flight control and warrant future morphing studies."

Line 193:
"It is important to highlight that further analytical and experimental studies are also necessary to incorporate the variable aerodynamic capabilities of each species, shoulder and tail range of motion and in vivo wing and tail shapes to definitively confirm the optimal phenotype(s) for static pitch stability."

Note that we also effectively account for a shifting neutral point in the Monte Carlo sensitivity analysis shown in Extended Data Fig. 3 although this is done by allowing up to 15% error in the CG metric. The variation accounted for in the relative static margin is visualized in Extended Data Fig. 4. This investigation showed that even randomly allowing for errors in each species' CG our analysis still returned the conclusion of evolutionary selection for the ability to maintain stable and unstable configurations. We added a sentence to address how this also captures an effective neutral point shift as follows:

Line 378:
"Note that this analysis is equivalent to having accounted for the same magnitude shift in the neutral point with a fixed CG as well."

2. Did the authors characterize the effect of the CG shift due to the wrist and elbow flexion as a function of the arm to hand wing ratio? Are the species tested represent a group of species where the hand wing is significantly smaller than the arm wing, and would the results change if the species tested were broader?

Thank you for highlighting this question, we agree that the arm to hand wing ratio provides important insight to the wing-only results. To address this question, we quantified the arm to hand wing ratio as the distance from the root to the wrist joint divided by the distance from the wrist joint to the wing tip. We found that our species' maximum arm to hand ratio ranges from 21% to 123% with the majority of species' ratios between 40-80%. We believe that this does an adequate job of covering the possible variation within species. We do find that $\Delta\overline{y_{CG}}_{wing}$ scales with the arm to hand wing ratio. Note that there is not a significant effect of arm to hand wing ratio of the overall shift in the full bird x_{CG} or z_{CG}. We have added a sentence to show this result into the center of gravity section as follows:

Line 119:
"The range due to elbow and wrist morphing ($\Delta\overline{y_{CG}}_{wing}$) was positively associated with the arm to hand wing ratio (Extended Data Table 1), such that birds with a longer hand wing than arm wing would have a reduced capacity to shift the CG of the wing (such as the swift)."

Other Comments

• I (the moment of inertia) is a function of time and flight configuration. What are the time scales for wrist and elbow extension during flying? Have the authors considered recommending a parametric oscillation model for the equations of motion? Such a model could affect the stability and maneuverability metrics

The time scales of wrist and elbow extension vary significantly across our investigated species and at this time our focus was to quantify the absolute capacity of inertial changes rather than define those used over time. Similarly, discussion of a model for dynamic oscillations is outside the scope of our study although this is an intriguing avenue for future studies.

• Can the authors justify the statement in lines 109-110?

This statement should not have been included in the manuscript as clearly the results go on to show that the elbow and wrist affects the stability characteristics which in turn will affect the dynamic response. We are grateful that this was highlighted. We apologize for this oversight and have removed this statement.

• Please label the view for Figure 1 e.

We have incorporated this suggestion into Figure 1 by adding two textboxes, one between 1c and 1e and the other between 1d and 1f.

• It is hard to understand the ROM from Figure 1g. What do the square brackets mean? Is the ROM normalized by the wingspan? Would you please clarify figure 1g

The brackets are the axes of each species' range of motion with the scaling specified on the pigeon-only range of motion located between 1d and 1f. The range is in absolute elbow and wrist joint angles in degrees. We apologize that this wasn't clear. To address this concern, we have added the axis labels and titles to the bottom left-hand range of motion and additional tick marks to clarify.

• The manuscript would benefit from a strong summary paragraph that highlights the main takeaways of the article.

We have added a final summary paragraph to capture the key takeaways as follows:

Line 204:
"In summary, our results revealed that elbow and wrist ROM had a small relative effect on the CG location and pitch inertial component, but had a substantial effect on the roll and yaw inertial components. Although the static margin varied within our investigated species, we found that the phenotypic optimum for maximum and minimum static margin allows the majority of species to transition between stable and unstable flight, suggesting the need for a complex flight control system. Collectively, investigating the inertial characteristics of flying birds throughout elbow and wrist extension and flexion brings us one step closer to establishing a fundamental theory to quantify and then evaluate avian manoeuvrability."

Referee #3:

A. Summary of the key results.

This manuscript explores the effects of wing morphing on the mass distribution of birds, and the consequent effects on stability and control. It does so by measuring the range of motion inherent in the wings of cadavers across a range of taxa, which it uses to inform modelling of the changes in centre of mass (CoM) and inertia tensor (I). The authors show that a realistic range of wrist and elbow motion has comparatively small effects on the longitudinal position of the CoM, but that shoulder flexion can affect CoM position substantially. Conversely, a realistic range of wrist and elbow flexion has large effects on the roll and yaw moment of inertia, but little effect on the pitch moment of inertia. A phylogenetically controlled analysis is used to analyse the evolution of maximally and minimally stable configurations in birds under an Ornstein Uhlenbeck model, leading the authors to conclude that birds have evolved to be able to switch between stable and unstable flight configurations. This is contrasted with the orthodoxy first introduced by Maynard Smith (1952), that birds became increasingly unstable through evolution.

> Thank you for your comments and suggestions. We believe that we have been able to address your major concerns by adjusting our analysis to account for your suggested changes. We are grateful for all the reviewers' suggestions as we believe that this has led to a substantial improvement of our manuscript.

B. Originality and significance

As the authors note, most research on avian flight stability focusses on aerodynamics, although the importance of mass distribution is well known, and was analysed for cadavers with fully spread wings (i.e. without morphing) by Thomas and Taylor (2001) in Ref. 12. That paper concluded "it seems reasonable to suggest that an animal that is statically stable during normal flight should not deliberately make itself unstable, except perhaps transiently during unsteady manoeuvres. … If it is found that flying animals do switch facultatively between stable and unstable flight, then this will surely represent one of the most remarkable capabilities of their locomotor control systems." That being so, the present paper's key conclusion that "evolutionarily pressures may be maintaining the ability to shift between stable and unstable configurations" is indeed a significant one – particularly given current interest in morphing-wing vehicles. I would encourage the authors to review the concluding paragraph of Ref 12, and particularly the text replaced by the ellipsis above, to see if they agree that there are implications for control reversal that may be worthy of further discussion. (Perhaps not during unsteady manoeuvres if morphing is used only transiently, but presumably so in steady manoeuvres if morphing is used to switch between stable versus unstable setpoints.)

> Thank you for this suggestion. We have adjusted two sentences in the introduction to better highlight the contribution and previous work as follows:
>
> Line 48:
> *"However, studies often overlook the equally essential inertial properties (Fig. 1a) or use static morphology approximations for individual species[13,17–20]."*
>
> Line 56:

"Physically shifting a bird's morphology will shift the CG but, it is not currently known how much the CG moves as a bird morphs."

We are grateful that you highlighted this key conclusion in Thomas and Taylor's fundamental paper, which we agree should be discussed in our conclusion. We have added two sentences to bring this to the forefront of the discussion and highlight some possibilities for future work as follows:

Line 186:
"Elbow and wrist flexion and extension alone offers birds the capacity to shift between these pitch stability modes but, if and when a flying bird does shift between these modes remains to be seen. As highlighted by Thomas and Taylor[12], dynamically switching between stable and unstable flight modes likely requires a bird to implement substantially different control algorithms and therefore switching between these modes would necessitate a complex flight control system. Future work is required to identify if and how the avian neurological system accounts for such distinct flight conditions."

We also believe that we should have better highlighted the results from Thomas and Taylor as that was one of the first studies to discuss the importance of inertial properties in stability as well as to highlight the possibility that modern birds are not unstable in pitch. Along with the new sentence in the introduction that is highlighted above (Line 48) we have adjusted another discussion sentence as follows:

Line 182:
"Although studies have suggested that modern birds may be capable of stabilized flight[12,13,15], it is widely believed that birds have evolved to be unstable in pitch to enhance manueverability[6]."

C.Data & methodology: validity of approach, quality of data, quality of presentation

The overall approach is appropriate to answering questions on the effects of varying mass distribution, but it seems a little surprising that the authors have not attempted to overcome the main limitation of their study that they identify (lines 183-5). Specifically, the stability metrics that they compute are based on the position of the CoM relative to the wing's aerodynamic centre (AC), but only take account of variation in the former. It is possible I have misunderstood the Methods on this point, but as the authors assume that the aerodynamic centre of the wing is located at its quarter-chord point, it would presumably be possible to model its movement with changes in wing position, based on the information they have captured on wing motion. (Note: line 152 refers this position to one quarter of the root chord, but this is unlikely to be accurate for a highly swept wing, for which I would have assumed that the mean chord would be the more accurate measure.) In principle, the main conclusion of the paper, that "evolutionarily pressures may be maintaining the ability to shift between stable and unstable configurations" would be stronger if the metric upon which this were based had been calculated to include the coupled motion of the CoM and AC during wing morphing. That said, the limitation is noted appropriately at the point that this conclusion is made.

Thank you for highlighting this concern, which was also raised by referee #2. We agree that the estimation of the neutral point should be better quantified. To address this concern, we leveraged the results from our previously published study on gull wing-body configurations to investigate which chord metric scaled with the estimated neutral point throughout elbow and wrist flexion and extension. We compared a total of 6 different metrics and found that the quarter chord point of the standard mean chord was the best at approximating the known neutral point for the gull wing-body configurations. We have detailed this analysis in a new supplementary document entitled "Supplemental methods: Aerodynamic approximations". As a result of this investigation, we have adjusted our analysis of stability and agility to incorporate this improved aerodynamically informed metric.

We are grateful that this concern was highlighted as we believe it improves the applicability of our results. As this chord metric was selected solely based on a rigid gull wing study, we have kept an adjusted version of our qualifying statement about how this work must be verified for other species as follows:

Line 193:
"It is important to highlight that further analytical and experimental studies are also necessary to incorporate the variable aerodynamic capabilities of each species, shoulder and tail range of motion and in vivo wing and tail shapes to definitively confirm the optimal phenotype(s) for static pitch stability."

However, we believe that the adjusted analysis with the mean chord metric will now better account for the aerodynamic implications of elbow and wrist morphing across all wings. Of note, this change affected two conclusions about the pitch agility: we found that the normalized agility metric now scales negatively with mass and that there is evidence of selection acting on the upper bound of agility. As agility is largely informed by this static margin, we selected to focus the discussion on evolutionary pressure on the static margin itself. To this end, our revised stability investigation provides evidence supporting the ability to transition between stable and unstable flight albeit with a stronger selection pressure towards stability. This difference was expected because the neutral point predicted with the standard mean chord is predominately more aft than that predicted from the root chord, but as the reviewer highlighted, this does give a more accurate estimation than the root chord. In all, we have created a new supplemental document and substantially revised the final five paragraphs of the paper as well as the methods section "Agility and stability metrics" to incorporate these changes.

The quality of the data is excellent: the species are well-chosen for comparative analysis, and the estimates of mass distribution appear to be well done, supported by a detailed description of the R package that the authors created to implement this. The graphical presentation of the figures is generally excellent, but symbols and abbreviations are not always explained in the legends and the very brief form of the legends makes it impossible to be certain what is being shown in some panels. I appreciate that Nature constrains figure legend lengths for papers with Methods sections, but in that case, the detail needs to be unambiguously provided in the Methods.

Thank you for your supportive comments and for highlighting that the figures could be clearer. We have gone through the abbreviations and symbols in each figure and adjusted to ensure that each was explained in either the figure legends or the figure itself and added additional supporting information into the methods section.

Figure 1:
We have added defined the abbreviation of moment of inertia in the legend. We have also added textboxes to clearly define the views of represented in 1c, d, e, and f. We have also added the axis labels and titles to the lower left-hand plot of panel 1g to improve legibility.

Figure 2:
We added specification of what origin was used to measure each center of gravity component to the legends of panels f and g. Within the legend, we made many adjustments for clarity as follows:

"***Figure 2. A bird's CG is minimally affected by elbow and wrist flexion and extension. a****, Time-calibrated phylogeny for 22 species (mya, million years ago). The elbow and wrist ROM (opaque polygons, convex hulls) affect (**b**) $\overline{x_{CG}}$ and $\overline{z_{CG}}$ (over bar indicates normalization by body length), and (**c**) $\overline{y_{CG}}_{wing}$(over bar indicates normalization by maximum half span). The CG range is overlaid with the maximum bounds due to*

*90° shoulder rotation (transparent polygons), which (**d**) increase with increasing ratio of wingspan to body length. **e**, Effect size (η^2) of elbow, wrist, and interaction on each CG component per specimen. The log-transformed mean values of (**f**) $\overline{x_{CG}}$, $\overline{z_{CG}}$ * (Extended Data Table 1) and (**g**) $\overline{y_{CG}}_{wing}$ did not scale with body mass as the phylogenetic generalized linear mixed model (PGLMM) did not differ significantly from the null slope. 95% confidence intervals visualized by transparent ribbons.*"

Figure 3:
We specified that the mean inertial components were log-transformed.

Figure 4:
Panels a and b were substantially revised, and we added words to call-out the agility and stability attributes relative to the *y* axis. We removed the body length measure between panels c and d as it was not necessary for understanding. Further, we adjusted the legend to clarify the symbols used in the figure as follows:

*"**Figure 4. Evolution selects for both pitch stability and instability, but modern birds exhibit highly variable pitch agility and stability characteristics.** We derived a pitch agility metric, (**a**) which highlights that heavier birds are less agile and **b**, have a reduced static margin range. An Ornstein Uhlenbeck model provided evidence of selection pressures acting on an unstable minimum (dashed line: min. θ_{sm}) and a stable maximum (dashed line: max. θ_{sm}) static margin, and $\overline{x_{CG}}$ (θ_{CG}). This $\overline{x_{CG}}$ position is (**c**) stable if it is in front of the NP and (**d**) unstable if it is behind the NP. **e**, The investigated species exhibited a wide variety of static margins and absolute normalized agility (right maximum, left minimum)."*

Extended Data Figure 2:
We adjusted the legend for improved clarity and less unexplained acronyms. Additionally, we improved the figure caption to clarify what was plotted and the implication of distributions as follows:

*"**Extended Data figure 2. A power analysis confirmed the validity of Ornstein Uhlenbeck (OU) models for three key traits.** We used a Monte Carlo-based method to investigate if our phylogeny provides support for the use of the OU model. This returns the distribution of likelihood ratios under each model for (**a**) $\overline{x_{CG}}$, (**b**) maximum static margin, and (**c**) minimum static margin. Comparing the likelihood ratios distributions produced under both a Brownian motion (BM) model (grey) and OU model (yellow) to the observed likelihood ratio (dashed black line) revealed that the OU model was a better fit for our three key traits (see methods)."*

Note that these amendments were done in addition to substantial revision of the methods section "Phylogenetic and statistical analyses" that provides additional quantification of the results and explanation.

D. Appropriate use of statistics and treatment of uncertainties

The use of statistics and treatment of uncertainties seems broadly appropriate, but the description of the model of trait evolution and the legends and axis labels for the accompanying figures seem somewhat incomplete. I would recommend considering review of this section by a specialist statistician, given that there are certain difficulties involved in the interpretation of these models – particularly at small sample sizes (see: Natalie Cooper, Gavin H. Thomas, Chris Venditti, Andrew Meade, Rob P. Freckleton, A cautionary note on the use of Ornstein Uhlenbeck models in macroevolutionary studies, Biological Journal of the Linnean Society, Volume 118, Issue 1, May 2016, Pages 64–77, https://doi.org/10.1111/bij.12701).

We apologize for not providing sufficient detail in our manuscript on these methods. We used the R package "pmc" to address the possible concerns within the smaller sample sizes as highlighted by Cooper et al by using a MCMC analysis to verify that the OU model was appropriate selection (Extended Data Fig. 2). However, this was previously not well described in the methods. To address this concern, we have adjusted the figures for clarity see above description of changes to Extended Data figure 2, substantially revised the relevant methods section "Phylogenetic and statistical analyses" and included the suggested citation. In particular the second paragraph of that methods section was entirely adjusted to address this concern as follows:

Line 343:
"Next, to investigate the phenotypic optimum of the agility and stability traits, we independently fit both Brownian motion (BM) and Ornstein Uhlenbeck (OU) models to the absolute data using the R package geiger[35]. We assumed that all species belong to the same regime and thus, fit single-peak evolutionary models. This analysis revealed that there was evidence that the OU model was a better model fit for all three of our selected traits ($\overline{x_{CG}}$, maximum and minimum static margin) due to a lower Akaike Information Criterion with correction for smaller sample sizes (AICc). Because of the smaller sample size of our study[36], we ran a Monte Carlo simulation (n=5000) with the R package pmc[37] to validate that selecting the OU model over the BM model was appropriate (Extended Data Fig. 2). This method returns a distribution of likelihood ratios (difference of the maximum likelihood estimates for each model) when the traits have been simulated n times under each model. These distributions are then compared to the observed likelihood ratio (black dashed vertical lines in Extended Data Fig. 2). For details refer to Boettiger et. al[37]. We found that the likelihood ratio predicted by a BM model was more extreme than the observed ratio for the minority of simulations ($\overline{x_{CG}}$: 0.2%, maximum static margin: 0.1%, minimum static margin: 1%). Further we had sufficient power to differentiate the two models as the majority of the simulations under the OU model fell outside of 95th percentile of the BM distribution ($\overline{x_{CG}}$: 73.8%, maximum static margin: 77.2%, minimum static margin: 67.2%). 95% confidence intervals were constructed for each reported metric of each trait (Extended Data Table 2). Together these results provide confidence that the observed likelihood ratio of each trait is more likely to occur under an OU model than a BM model."

Currently, the authors' main conclusions seem to be relying quite heavily on model estimates of what appear to be weak but robust selection pressures. It would therefore be reassuring to report more fully on the actual measured distribution of the maximum and minimum stability ratios. I think this may be what is shown in Fig. 4e, but attractive as this panel is, it is not entirely clear whether it is supposed to show the results of measurements or the model, and it would be useful to support this with a basic plot of the data.

Ultimately, unless a large majority of the species are shown empirically to have a range of CoM motion that allows them to transition between stable and unstable configurations, the main conclusion of the manuscript is heavily dependent on the results of a somewhat opaque model of evolution. I'd therefore encourage adding some basic statistics on the median stability/instability of the maximum and minimum configurations, which are relevant anyway to understanding how general the authors' findings may be across extant birds.

We agree that the results in the current birds tested should be better presented. To this end, we have added a sentence to clarify how many species in our study do or do not show the ability to shift across the modes as follows:

Line 166:
"We found that four species were entirely stable, one species was entirely unstable, and 17 species had the capacity to shift between stable and unstable flight (Fig. 4b and e)."

We directly incorporated the reviewer's suggestion to directly plot the maximum and minimum static margin as well as added dashed lines to specify the location of the calculated maximum and minimum optimal phenotypes (Fig. 4b). This allowed us to more explicitly indicate that it was the true data that are used to create Fig. 4e. Finally, we substantially adjusted the caption to improve the clarity of the explanation used in Fig. 4e as follows:

*"**Figure 4. Evolution selects for both pitch stability and instability, but modern birds exhibit highly variable pitch agility and stability characteristics.** We derived a pitch agility metric, (**a**) which highlights that heavier birds are less agile and **b**, have a reduced static margin range. An Ornstein Uhlenbeck model provided evidence of selection pressures acting on an unstable minimum (dashed line: min. θ_{sm}) and a stable maximum (dashed line: max. θ_{sm}) static margin, and $\overline{x_{CG}}$ (θ_{CG}). This $\overline{x_{CG}}$ position is (**c**) stable if it is in front of the NP and (**d**) unstable if it is behind the NP. **e**, The investigated species exhibited a wide variety of static margins and absolute normalized agility (right maximum, left minimum)."*

I am not sure whether I am reading Extended Data Figure 1 correctly, which reflects the fact that the legend needs some improvement. However, on the face of it, this figure seems to show a bias in the Brownian model (which seems rather surprising), and an extremely small difference between the observed maximum and minimum stability ratios in comparison to the range of variation in the Monte Carlo analysis (much smaller than the Results seem to report). I therefore reserve judgement on this analysis pending fuller description in a revised Methods and Results section, and fuller elaboration of the figure legends, etc.

Thank you for highlighting that this description was not clear. We have addressed this concern by including a new detailed explanation of the MCMC method used to generate this figure as detailed above in "Phylogenetic and statistical analyses" as follows:

Line 343:
"Next, to investigate the phenotypic optimum of the agility and stability traits, we independently fit both Brownian motion (BM) and Ornstein Uhlenbeck (OU) models to the absolute data using the R package geiger[35]. We assumed that all species belong to the same regime and thus, fit single-peak evolutionary models. This analysis revealed that there was evidence that the OU model was a better model fit for all three of our selected traits ($\overline{x_{CG}}$, maximum and minimum static margin) due to a lower Akaike Information Criterion with correction for smaller sample sizes (AICc). Because of the smaller sample size of our study[36], we ran a Monte Carlo simulation (n=5000) with the R package pmc[37] to validate that selecting the OU model over the BM model was appropriate (Extended Data Fig. 2). This method returns a distribution of likelihood ratios (difference of the maximum likelihood estimates for each model) when the traits have been simulated n times under each model. These distributions are then compared to the observed likelihood ratio (black dashed vertical lines in Extended Data Fig. 2). For details refer to Boettiger et. al[37]. We found that the likelihood ratio predicted by a BM model was more extreme than the observed ratio for the minority of simulations ($\overline{x_{CG}}$: 0.2%, maximum static margin: 0.1%, minimum static margin: 1%). Further we had sufficient power to differentiate the two models as the majority of the simulations under the OU model fell outside of 95th percentile of the BM distribution ($\overline{x_{CG}}$: 73.8%, maximum static margin: 77.2%, minimum static margin: 67.2%). 95% confidence intervals were constructed for each reported metric of each trait (Extended Data Table 2). Together these results provide confidence that the observed likelihood ratio of each trait is more likely to occur under an OU model than a BM model."

Additionally, we have adjusted the labelling of this figure for clarity as follows:

*"**Extended Data figure 2. A power analysis confirmed the validity of Ornstein Uhlenbeck (OU) models for three key traits.** We used a Monte Carlo-based method to investigate if our phylogeny provides support for the use of the OU model. This returns the distribution of likelihood ratios under each model for (**a**) $\overline{x_{CG}}$, (**b**) maximum static margin, and (**c**) minimum static margin. Comparing the likelihood ratios distributions produced under both a Brownian motion (BM) model (grey) and OU model (yellow) to the observed likelihood ratio (dashed black line) revealed that the OU model was a better fit for our three key traits (see methods)."*

E. Conclusions: robustness, validity, reliability

The key limitation of this paper is that the method appears to take account only of the movement of the CoM as the wings flex, and not of coupled movement of the AC. This seems a surprising omission given the authors' statement (line 78) that their own "previous studies have shown that this range of motion (ROM) allows a substantial shift in the neutral point" (where the neutral point is equated with the AC). In fact, I am left wondering whether this reflects an omission in the description of the Methods, rather than an omission in the analysis, and suggest that the authors revise lines 268-271 for clarity. That said, it seems unlikely that taking account of coupled AC motion would fundamentally alter the conclusions, because the AC and CoM are likely to move fore and aft together. Even so, the conclusions would be much more robust if this were analysed.

We agree and are grateful that this limitation was highlighted. We have incorporated this analysis as detailed above. In all, this led to a new supplemental document and substantial revisions to the final five paragraphs of the paper as well as to the methods section "Agility and stability metrics".

I am also not entirely convinced by the conclusion "Our results indicate that it is unlikely that flight dynamics during elbow and wrist flexion and extension will be substantially affected by a shifting CG." This conclusion seems to be based on the result that the CoM shifts by a maximum of 3% of body length under realistic flexion of the wrist and elbow. However, as the rest of the paper shows, the length of the body is not the relevant measure, because (i) statically stable systems become statically unstable in a threshold manner; and (ii) what therefore matters is the movement in relation to the static margin (i.e. the difference between the position of the CoM and AC as a fraction of the chord). I'd suggest restating the results accordingly, with appropriate normalisation.

It is also not clear to me how the conclusion that CoM movement during elbow/wrist flexion has little effect on flight dynamics relates to the authors' main conclusion that birds have evolved to sit at the threshold of stability versus instability. Presumably, this is because the maximum versus minimum stability measures take account of either the much larger effect of shoulder motion, or a wider range of elbow/wrist motion? If so, this needs clarification in the text, as the definition of the maximum and minimum stability metrics does not appear to be clearly stated in the text.

This statement should not have been included in the manuscript as clearly the results go on to show that elbow and wrist affects the stability characteristics which in turn will at least somewhat affect the dynamic response. We are grateful that this was highlighted. We apologize for this oversight and have removed this statement.

F. Suggested improvements: experiments, data for possible revision

Besides the major comments above, I would suggest the following detailed revisions:

(i) The main text at line 167 and following refers to a "log-transformed static pitch stability ratio", which I assume is the same as the "log transformed static margin ratio" defined at Eq. 7. Please make the terminology consistent, and if using the "static margin" terminology, then note that this jargon is not defined in the paper.

We apologize for the inconsistent and confusing terminology, due to the improvements in the neutral point estimation we have not used any stability ratio and instead have adjusted to solely refer to the maximum (most positive such that NP is behind of CG) and minimum (most negative such that NP is ahead of CG) static margin.

(ii) There is no logarithm shown in Eq. 7, which makes the meaning of "log-transformed" and the data to which this refers rather ambiguous. I assume the authors mean that they log-transformed Eq. 7. If so, they should show this, and confirm the base of the logarithm, which is needed for the numerical results of the OU model to be interpretable.

We agree that this was confusing and apologize for this. In our original submission we had been using the log-transformed values in the evolutionary fits. However, we have now adjusted to use the non-transformed stability data instead to avoid confusing terminology and clarify the analysis. Further, to improve the interpretability of the OU model outputs we plotted the optimal phenotypes as black dashed lines in Fig. 4b, which can now be directly contrasted to the results of each individual in our study.

(iii) The Methods need further clarification throughout, particularly in relation to the calculation of the position of the AC.

To address this concern, we have substantially revised the majority of the methods and created a new supplementary document to detail the methods used to approximate the aerodynamic centre.

(iv) The definitions of manoeuvrability (line 26) and especially agility (line 64) are stated rather imprecisely, and I would struggle to quantify either without further explanation.

Although we agree that the definitions of maneuverability and agility introduced in these sections are very broad, we believe that this is the most comprehensive definition that avoids constraining these metrics arbitrarily. However, we agree that we should provide a more precise statement for our developed pitch agility metric within the main text and have adjusted a sentence as follows:

Line 149:
"*Using these results, we derived a novel normalized pitch agility metric that is proportional to the angular acceleration about the y axis due to a change in the angle of attack (a form of torsional agility, Fig. 1a, see methods).*"

(v) It would help to use bold font to show that Eqs. 1-2 are matrix equations, particularly having said that there are 6 of these equations.

Agreed. We have incorporated this suggestion by adding arrows to the vectors and bolding the tensor.

(vi) The definition of static stability at line 66 as "the inherent tendency to return to an initial equilibrium after a disturbance" reads more like a definition of dynamic stability. I would say "the initial tendency to return towards equilibrium after a disturbance".

Agreed. We have incorporated this suggestion into this sentence directly.

(vii) At line 73 "across all avian clades" rather neglects the fact that a clade can be defined at any level within a phylogeny. I know what the authors are trying to say, but clade needs some qualification here in terms of taxonomic level.

Agreed. We have adjusted the relevant sentence to avoid unnecessarily binning the investigated species as follows:

Line 72:
*"To determine how the inertial characteristics vary during wing morphing, we developed a general analytical method to quantify any flying bird's CG and **I**, and used a comparative analysis to investigate 22 species spanning the phylogeny defined by Prum et al.[21] except for Palaeognathae as this clade contains largely flightless birds."*

(viii) Lines 85-97 describe validation of the method against old data. Whilst useful, I doubt that most readers will be interested in the detail, and suggest moving this to Methods.

Agreed. We have relocated this paragraph into a new section in the methods entitled "Validating AvInertia".

(ix) Line 93 "encapsulated" should probably be "encompassed", or the sentence could be restructured to describe this in terms of intervals.

Agreed. We have incorporated this suggestion directly.

(x) At line 158 and in Fig. 4 "agility capacity" is used as if this is a precise quantity, but the meaning of the term is nowhere defined. I assume this is the "pitch agility metric" defined at Eq. 3 in the Methods, but again, the terminology needs to be consistent.

We apologize for not defining this metric. We had intended it to refer to the pitch agility metric once normalized. We have removed this terminology and solely use "normalized pitch agility" to avoid any confusion.

(xi) Some figure legends need expansion to make the figures understandable, and some axes are missing labels or units (e.g. Extended Data Figure 1).

We have reviewed and updated each figure to ensure all figures have been clarified. Details specifying each change to the figures has been provided above.

(xii) At Line 269, I can't parse the phrase "the neutral point is proportional to the wing's theoretical aerodynamic centre".

We have entirely revised this section following the improvement of the neutral point estimation and have removed this sentence.

(xiii) It is not completely clear how the maximum and minimum stability ratios are defined. There needs to be a clear statement somewhere of the wing configurations to which these refer, in terms of which joints and flexing, and through what angle(s).

We agree this was not as clear as it should have been. We have added a new extended data figure (Extended Data Fig. 1) that identifies which configurations have the maximum and minimum static margin for each species.

F. References: appropriate credit to previous work?

Suggest considering the following:

Hedrick TL, Usherwood JR, Biewener AA. Wing inertia and whole-body acceleration: an analysis of instantaneous aerodynamic force production in cockatiels (Nymphicus hollandicus) flying across a range of speeds. J Exp Biol. 2004 Apr;207(Pt 10):1689-702. doi: 10.1242/jeb.00933. PMID: 15073202. [Detailed measurements of wing inertia.]

Mills, R., Hildenbrandt, H., Taylor, G.K., Hemelrijk, C.K. (2018). Physics-based simulations of aerial attacks by peregrine falcons reveal that stooping at high speed maximizes catch success against agile prey. PLoS Comput. Biol.14(4), e1006044. https://doi.org/10.1371/journal.pcbi.1006044. [Modelling of variation in roll inertia during manoeuvres].

Cheney JA, Stevenson JPJ, Durston NE, Maeda M, Song J, Megson-Smith DA, Windsor SP, Usherwood JR, Bomphrey RJ. Raptor wing morphing with flight speed. J R Soc Interface. 2021 Jul;18(180):20210349. doi: 10.1098/rsif.2021.0349. Epub 2021 Jul 14. PMID: 34255986; PMCID: PMC8277465. [Recent work probably post-dating submission].

We have added the first two citations to a sentence within the introduction as follows:

Line 48:
"However, studies often overlook the equally essential inertial properties (Fig. 1a) or use static morphology approximations for individual species[13,17–20]*."*

We had difficulty finding the best place to cite the final citation given that (excellent) paper's focus on morphology (without direct reference to inertial properties).

G.Clarity and context: lucidity of abstract/summary, appropriateness of abstract, introduction and conclusions.

The writing is mostly clear, but there are some inconsistencies in terminology between the Methods and Main Text (see above), and points where the language would benefit from some tightening up for precision (see above). In places, the text seems aimed at a more specialist audience than the readership of Nature, notably the section on model validation.

We have kept these suggestions in mind while reviewing the entire manuscript. In addition to the specified changes made to the above concerns, we hope the revised manuscript as a whole addresses these concerns.

Reviewer Reports on the First Revision:

Referee #1 (Remarks to the Author):

The authors have considered all the referees comments carefully and thoughtfully - the manuscript is much improved. My own concerns have been addressed adequately although I am still left hungry for a further analysis including movements of the shoulder. I look forward to reading this in a subsequent paper. I agree with Reviewer 3 that the recent Cheney paper is relevant here, since it reports in vivo morphing of both area (fig 6) and shape (fig 10) consistent with skeletal articulation of the wings. Having said that, these changes are rather small, probably because the birds they discuss were gliding at different speeds and not doing much by way of manoeuvring.

Referee #2 (Remarks to the Author):

The authors have adequately addressed my concerns. I believe the changes significantly improved the overall paper quality and strengthened the analysis and resulting conclusions.

I would like to congratulate the authors on a well-written manuscript and on the significant work that they have put into improving the manuscript.

Referee #3 (Remarks to the Author):

The authors have made substantial improvements to their manuscript in response to feedback from the reviewers. Specifically, they have revised their method to address what was a significant omission identified by reviewers 2 and 3, and now model the aerodynamic effect of wing morphing on the location of the neutral point. They have not modified their method to address two further limitations identified by reviewers 1 and 2, who recommended modelling the effects of shoulder and tail motion. I accept that it would not be practical for the authors to do so this without significant experimental work, and that the existing results on the effects of elbow and wrist motion are useful in themselves. Even so, this limits the strength of the conclusions that can be drawn, and the revised method has produced some significant changes to the conclusions regarding agility (compare Fig. 4e between versions), which I think call into question the robustness of this metric. Some more detailed comments follow.

1. The central result of the manuscript has been substantially strengthened, and can now be robustly stated as: “the measured range of wrist and elbow motion alone is sufficient to enable switching between stable and unstable flight in 18 out of 22 species of bird”. As reviewers 1 and 2 notes, anatomical coupling of shoulder motion to wrist and elbow motion could modify this conclusion, as

could any accompanying motions of the tail. As tail motions are not anatomically coupled to wing motions, the lack of shoulder motion modelling is probably the greater limitation, but this reasoning does suggest an alternative interpretation of the authors' central result. Specifically, rather than reflecting selection to enable a transition between stable and unstable flight as currently argued, is it not possible that the range of movement of the wing's neutral point reflects selection to enable the tail to support the high positive lift needed to facilitate slow flight?

2. Whereas the results related to the range of static margin of the wings appear robust (but see previous comment), the results related to agility have change markedly between the two versions of the manuscript. It is possible that the more recent results appropriately correct the earlier ones, but I am uneasy with the extent to which two similar metrics that ostensibly set out to measure the same performance criterion have arrived at such different conclusions. On close inspection, I think that there are clear reasons to doubt the validity of the metric used. For instance, Fig. 4e shows that the Lady Amherst's pheasant (a ground-dwelling species that rarely flies), has much higher pitch agility than a Cooper's hawk (an aerial hunter renowned for its agility) or raven (famous for its agility as one of the only birds capable of inverted flight). I doubt that ornithologists will find this result credible, particularly given the marked differences in agility claimed for the other species of pheasant and other species of hawk, and my feeling is that this is likely to point to a flaw in the metric that the authors have used to quantify agility. The most likely explanation, noting the very high agility stated for the storm petrel in this version of the manuscript, may be that this is attributable to some scaling artefact. However, the new agility metric also appears to treat stable and unstable configurations equally, which may be problematic if the high pitch acceleration that the metric is supposed to measure is associated with a return to equilibrium. My strong advice would be to remove the agility metric altogether, and to focus exclusively on the robust results relating to static margin.

3. The method that the authors have introduced to account for the aerodynamic effects of wing morphing (see Supplemental Methods) relies heavily on an empirical fit to a set of previous results from gull wings. For my own part, I would have begun from the principled assumption that the mean quarter chord point ought to provide the best estimate of the position of the wing-body neutral point (because this is what a blade element modelling approach would predict), and would have validated this assumption using a linear regression on the gull data. Instead, the authors have proposed six alternative metrics that they have fitted nonlinearly to the gull data, ultimately concluding that the neutral point varies as the 0.8 power of the mean quarter chord point. The confidence interval for this exponent needs to be treated with some scepticism, as the datapoints are presumably not independent, and the y-on-x regression method will in any case tend to bias the estimate below one. However, even if the exponent were genuinely less than one, I find it hard to imagine why this should be anything other than a local property of gull wings, so cannot see why this exponent should be expected to be applicable across a broader dataset of 22 species with varied flight morphology. [Please also cross-check Eqs. 5 and 6 of Supplemental Methods with Eq. 3 of the main methods, as there appears to be a mathematically important inconsistency in the use of brackets and absolute quantities.]

4. Close inspection of the agility metrics and revised static margins shows some quite substantial differences between measurements from different individuals of the same species, which are larger in some cases than the variation between species. This merits some discussion. Inter-individual variation also requires careful handling within the context of a phylogenetic model with branch lengths included, which also merits some discussion.

Author Rebuttals to First Revision:

Referee #1:

The authors have considered all the referees comments carefully and thoughtfully - the manuscript is much improved. My own concerns have been addressed adequately although I am still left hungry for a further analysis including movements of the shoulder. I look forward to reading this in a subsequent paper.

Thank you for your supportive comments and time spent reviewing our manuscript. We are grateful to all the referees as we agree that this review process has led to substantial improvements in our work. We are also excited to explore and discuss the role of the shoulder in future work.

I agree with Reviewer 3 that the recent Cheney paper is relevant here, since it reports in vivo morphing of both area (fig 6) and shape (fig 10) consistent with skeletal articulation of the wings. Having said that, these changes are rather small, probably because the birds they discuss were gliding at different speeds and not doing much by way of manoeuvring.

Thank you for highlighting this point. We have added this citation into the introduction as follows:

Line 81:

"*In addition, we assumed that the shoulder was set to allow a comparable wing orientation (see methods) and that the tail is furled, but these degrees of freedom play an important role in avian flight control*[22] *and warrant future morphing studies.*"

Referee #2:

The authors have adequately addressed my concerns. I believe the changes significantly improved the overall paper quality and strengthened the analysis and resulting conclusions.

I would like to congratulate the authors on a well-written manuscript and on the significant work that they have put into improving the manuscript.

Thank you for your supportive comments and time spent reviewing our manuscript. We are grateful to all the referees as we agree that this review process has led to substantial improvements in our work.

Referee #3:

The authors have made substantial improvements to their manuscript in response to feedback from the reviewers. Specifically, they have revised their method to address what was a significant omission identified by reviewers 2 and 3, and now model the aerodynamic effect of wing morphing on the location of the neutral point. They have not modified their method to address two further limitations identified by reviewers 1 and 2, who recommended modelling the effects of shoulder and tail motion. I accept that it would not be practical for the authors to do so this without significant experimental work, and that the existing results on the effects of elbow and wrist motion are useful in themselves. Even so, this limits the strength of the conclusions that can be drawn, and the revised method has produced some significant changes to the conclusions regarding agility (compare Fig. 4e between versions), which I think call into question the robustness of this metric. Some more detailed comments follow.

Thank you for your supportive comments and time spent reviewing our manuscript. We are grateful to all the referees as we agree that this review process has led to substantial improvements in our work. Please find detailed responses to your comments below.

1. The central result of the manuscript has been substantially strengthened, and can now be robustly stated as: "the measured range of wrist and elbow motion alone is sufficient to enable switching between stable and unstable flight in 18 out of 22 species of bird". As reviewers 1 and 2 notes, anatomical coupling of shoulder motion to wrist and elbow motion could modify this conclusion, as could any accompanying motions of the tail. As tail motions are not anatomically coupled to wing motions, the lack of shoulder motion modelling is probably the greater limitation, but this reasoning does suggest an alternative interpretation of the authors' central result. Specifically, rather than reflecting selection to enable a transition between stable and unstable flight as currently argued, is it not possible that the range of movement of the wing's neutral point reflects selection to enable the tail to support the high positive lift needed to facilitate slow flight?

We are grateful that the reviewer has highlighted this exciting interpretation, we entirely agree that this is another conclusion that can be drawn from our results. As pointed out by the reviewer shifting the neutral point in front of the CG will allow for a bird to attain trimmed/equilibrium flight when the tail is generating a positive lift force, although still resulting in a shift from a stable to unstable mode. As Usherwood et al. showed that gliding raptors use positive tail lift in slow gliding flight this points to an exciting connection between the two results. We have incorporated this point (including this new citation) within the conclusion with two new sentences as follows:

Line 182:

"Further, our findings offer insight on how birds perform slow glides with positive tail lift[29]. By maintaining the capacity to relocate the wing-body NP in front of the CG, birds may achieve an equilibrium, albeit unstable, flight condition."

In addition, we have included a new sentence within the supplemental materials:

Supplemental methods – Aerodynamics; Section 2, Page 4:

"As with our key results, this could additionally suggest that birds have the ability to shift their neutral point in front of their centre of gravity to balance the positive tail lift that is required for weight support in slow gliding flight."

In addition, we found your statement of our finding to be especially powerful and succinct, and have included it in the final summary paragraph as follows:

Line 197:

"Although inter- and intra-specific variation was apparent, we found that the measured range of wrist and elbow motion alone is sufficient to enable switching between stable and unstable flight in 17 out of 22 bird species."

2. Whereas the results related to the range of static margin of the wings appear robust (but see previous comment), the results related to agility have change markedly between the two versions of the manuscript. It is possible that the more recent results appropriately correct the earlier ones, but I am uneasy with the extent to which two similar metrics that ostensibly set out to measure the same performance criterion have arrived at such different conclusions. On close inspection, I think that there are clear reasons to doubt the validity of the metric used. For instance, Fig. 4e shows that the Lady Amherst's pheasant (a ground-dwelling species that rarely flies), has much higher pitch agility than a Cooper's hawk (an aerial hunter renowned for its agility) or raven (famous for its agility as one of the only birds capable of inverted flight). I doubt that ornithologists will find this result credible, particularly given the marked differences in agility claimed for the other species of pheasant and other species of hawk, and my feeling is that this is likely to point to a flaw in the metric that the authors have used to quantify agility. The most likely explanation, noting the very high agility stated for the storm petrel in this version of the manuscript, may be that this is attributable to some scaling artefact. However, the new agility metric also appears to treat stable and unstable configurations equally, which may be problematic if the high pitch acceleration that the metric is supposed to measure is associated with a return to equilibrium. My strong advice would be to remove the agility metric altogether, and to focus exclusively on the robust results relating to static margin.

We agree that change in the evolutionary interpretations of the agility metric between the revisions suggests reduced support for any evolutionary constraints on agility. For this reason, we reframed our previous revision to focus solely on the robust static margin evolutionary results. We would like to clarify that the absolute values of the agility metric did not shift substantially in an absolute sense between the revisions. It is possible that we should have been clearer with respect to the aesthetic changes made to the agility plot in Fig. 4. Fig. 4b from the first submission had a logarithmic scaled y-axis and showed the absolute maximum agility (i.e. the maximum magnitude between the unstable and stable maximum that is now plotted). Per the previous review comments, we agree that this previous version was confusing and too derived. Thus, Fig. 4a from the second submission showed the exact maximum and minimum normalized pitch agility metric plotted on an absolute *y*-axis scale.

Nonetheless, we agree with the reviewer that our normalized agility metric is still too derived for the audience and could possibly be misinterpreted. We understand the reviewer's concern with the scaling artifact that may be introduced by scaling our results to obtain a "nondimensional pitching rate" as is done in traditional aircraft studies. We agree that interpreting this metric requires special caution and may just confuse readers and thus detract from our major results. To this end, we have replaced the normalized pitch agility metric with the dimensional pitch agility metric at all relevant sections. This holds the physically expected and consistently returned result that absolute agility decreases with increasing size and speed (and thus mass as both parameters scale positively with mass). As we have broadly defined pitch agility in our work to be the ability to generate an angular acceleration, we do believe that displaying the stable configuration results still holds value, but as the reviewer points out this could be confusing and should be directly highlighted for the reader. To account for these changes, we have revised multiple sections of the manuscript as follows as well as adjusted Fig. 4 and Extended Data Fig. 4.

Line 141:
"We next asked if inertial characteristics can be used to estimate a bird's pitch agility. However, because both inertia and aerodynamics are fundamental to flight dynamics, we first used aerodynamic theory and data from a rigid gull wing[16] to obtain an estimate for the NP, and thus the static margin for each configuration (see methods and electronic supplementary materials). Using these results, we derived a novel pitch agility metric that is proportional to the angular acceleration about the y axis due to a change in the angle of attack (a form of torsional agility, Fig. 1a, see methods). Note that agility in a stable configuration indicates that the developed acceleration would tend to return the bird towards an equilibrium position. We found that the pitch agility range decreases as body mass increases, which was expected because flight speed and body size scale positively with mass[26] (Fig. 4a, Extended Data Table 1). These results are further driven by the static margin whose range also decreases as mass increases (Fig. 4b, Extended Data Table 1). Incorporating the shoulder joint ROM would broaden the static margin range because the resultant

NP shift is likely larger than the CG shift as evidenced by morphing UAVs with shoulder-inspired joints[27,28].”

Methods:
We have removed the sentences at Line 420 that explained the normalization procedure.

Further, we believe that we should be more cautious with the word “agility”, which often brings to mind lateral maneuvers such as the transition to inverted flight. Our work, unfortunately, cannot comment on avian lateral agility which possibly differs substantially from pitch maneuvering capabilities. To ensure clarity, we adjusted all references within the manuscript to our agility metric to specifically state “pitch agility” and have adjusted a sentence in the conclusion to better highlight the need for lateral studies as follows:

Line 191:
“Future work is also required to extend this analysis into the roll and yaw axes to discuss lateral agility and stability, which will need to account for aerodynamic and inertial coupling[5].”

3. The method that the authors have introduced to account for the aerodynamic effects of wing morphing (see Supplemental Methods) relies heavily on an empirical fit to a set of previous results from gull wings. For my own part, I would have begun from the principled assumption that the mean quarter chord point ought to provide the best estimate of the position of the wing-body neutral point (because this is what a blade element modelling approach would predict), and would have validated this assumption using a linear regression on the gull data. Instead, the authors have proposed six alternative metrics that they have fitted nonlinearly to the gull data, ultimately concluding that the neutral point varies as the 0.8 power of the mean quarter chord point. The confidence interval for this exponent needs to be treated with some scepticism, as the datapoints are presumably not independent, and the y-on-x regression method will in any case tend to bias the estimate below one. However, even if the exponent were genuinely less than one, I find it hard to imagine why this should be anything other than a local property of gull wings, so cannot see why this exponent should be expected to be applicable across a broader dataset of 22 species with varied flight morphology.

Thank you for highlighting this concern, we agree that there is inherent uncertainty caused by an empirical data fit. As the reviewer mentioned, we also began our investigation from the analytical result that the quarter chord would be a good selection based on thin airfoil theory and sought to validate our assumption by fitting a linear regression on the log-transformed data. Further, because there are many ways within the aeronautical literature that are used define a single chord metric for a complex 3D wing shape, we decided to leverage our existing results from the gull. To clarify our logic when approaching this analysis, we have revised a paragraph in the supplemental materials as follows:

Supplemental methods – Aerodynamics; Section 1, Page 2:
“Our approach was informed by traditional aerodynamic theory which predicts that the aerodynamic centre of a 2D thin airfoil will be at the quarter-chord location[5], this work can be extended to lifting line theory for steady flight conditions (commonly used in gliding flight) or blade element theory for revolving airfoils (commonly used in flapping flight). However, 2D thin airfoil approximations do not hold for the thick airfoils known to be used on the proximal wing sections of bird wings[6] nor for 3D wing shapes[5]. Although, advances in analytical methods have resulted in mathematical relationships that account for constant taper ratios or sweep[7,8], little information exists for wing shapes as complex as bird wings who have substantial and often nonlinear distributions of geometric twist, taper, sweep, and dihedral. Therefore, we investigated six different chord-based metrics[2,8,9] to establish which would best approximate the neutral point. The origin for all metrics within this study was set at the shoulder joint.”

To address the reviewer’s concerns related to the uncertainty of the empirical datafit, we performed a sensitivity analysis and recalculated the key optimal phenotypes while varying the exponent from 0.7 to 1.1. We found that our results remain robust. With this additional information and because the gull data is the only dataset to date which has quantified the neutral point of avian wing shapes across the full range of elbow

and wrist extension and flexion, we selected to continue with 0.8 as the exponent within our analysis. We have included this analysis as a new paragraph within the Supplemental Methods – Aerodynamics.

Supplemental methods – Aerodynamics; Section 1, Page 3:

> *"We verified that our key static margin findings were not substantially affected by the selected exponent by performing a sensitivity analysis. We found that despite varying the exponent from 0.7 to 1.1, all the optimal phenotypes for maximum static stability were stable whereas the optimal phenotypes for minimum static stability were unstable. Specifically, as the scaling parameter increased, we found that the optimal phenotype for the maximum static margin shifted from 31% to 15% of the maximum root chord and the minimum static margin shifted from -13% to -19%. Thus, increasing the exponent effectively serves to shift the results towards instability but our evolutionary findings remain supported. As the gull represents the only species with which we are able to estimate the relationship between geometric parameters and the neutral point across the full range of flexion and extension, we have selected to proceed with the gull-informed exponent of 0.8.*
>
> *In all, this result provides insight into the aerodynamic implications of morphing however this was informed by an aerodynamic analysis of only one species for rigid wing-body configurations. It will be important for future studies to account for inter-specific differences as well as a neutral point shift due to flexibility and porosity. In addition, we assumed that pitching moment caused by the lift force dominates the resultant moment similar in a manner similar to traditional aerodynamic analyses, however this work should be extended in the future to account for the effect of drag as well."*

In addition, we added a sentence in the methods to direct readers to the supplemental materials for this supplemental analysis as follows:

Line 409:

> *"In addition, we performed a sensitivity analysis on the exponent (see electronic supplementary materials)."*

[Please also cross-check Eqs. 5 and 6 of Supplemental Methods with Eq. 3 of the main methods, as there appears to be a mathematically important inconsistency in the use of brackets and absolute quantities.]

Thank you for highlighting this inconsistency. Our apologies, we should have put brackets around the x/c component in equation 6 in the supplemental methods and have updated this equation accordingly.

4. Close inspection of the agility metrics and revised static margins shows some quite substantial differences between measurements from different individuals of the same species, which are larger in some cases than the variation between species. This merits some discussion. Inter-individual variation also requires careful handling within the context of a phylogenetic model with branch lengths included, which also merits some discussion.

We agree that our results show evidence of intra-specific variation that will be exciting for future studies to investigate. First, to address this specific area of possible variation within our results, we have revised the methods to directly state that intra-specific variation on the final species mean has been accounted for within our sensitivity analysis as follows:

Line 466:

> *"To this end, we used a custom bootstrapping code (n=5000) and randomly sampled (with replacements) from each specimen's CG error range used for the sensitivity analysis to recalculate the mean value of the minimum and maximum static margin for each species."*

Line 472:

"Note that this analysis is equivalent to both accounting for the same magnitude shift in the NP with a fixed CG as well as accounting for possible inter-specific variation within the error bounds shown in Extended Fig. 4."

Finally, we have revised two sentences in the conclusion as follows:

Line 186:

"It is important to highlight that further work is required to incorporate the inter- and intra-specific aerodynamic capabilities, shoulder and tail ROM, and in vivo configurations to definitively confirm the optimal phenotype(s) for static pitch stability."

Line 197:

"Although inter- and intra-specific variation was apparent, we found that the measured range of wrist and elbow motion alone is sufficient to enable switching between stable and unstable flight in 17 out of 22 bird species. Further, an evolutionary analysis showed that the phenotypic optimum maximum and minimum static margin supports the ability to transition between stable and unstable flight, suggesting the need for a complex flight control system."

Reviewer Reports on the Second Revision:

Referee #3 (Remarks to the Author):

The authors have addressed all of my comments on their previous revision, and those of the other reviewers. I am satisfied with their responses to these, and congratulate them on their work. It occurs to me that if the authors feel it would be useful to include the figure showing the normalized version of their pitch agility metric as Supplementary Material then it would be reasonable to do so, as a complement to the figure in the main text. As discussed, the figure in the main text is less open to misinterpretation, but the normalized metric may still be useful in partitioning out the scale effects. But I leave that entirely up to them!

Author Rebuttals to Second Revision:

Referee #3:

The authors have addressed all of my comments on their previous revision, and those of the other reviewers. I am satisfied with their responses to these, and congratulate them on their work. It occurs to me that if the authors feel it would be useful to include the figure showing the normalized version of their pitch agility metric as Supplementary Material then it would be reasonable to do so, as a complement to the figure in the main text. As discussed, the figure in the main text is less open to misinterpretation, but the normalized metric may still be useful in partitioning out the scale effects. But I leave that entirely up to them!

Thank you for your supportive comments and time spent reviewing our manuscript. We have elected not to include the normalized metric to avoid distracting from the key messages in this work.
